# Angiopoietin-2 blockade suppresses growth of liver metastases from pancreatic neuroendocrine tumors by promoting T cell recruitment

**DOI:** 10.1172/JCI167994

**Published:** 2023-10-16

**Authors:** Eunhyeong Lee, Sophie O’Keefe, Alessandra Leong, Ha-Ram Park, Janani Varadarajan, Subrata Chowdhury, Shannon Hiner, Sungsoo Kim, Anahita Shiva, Richard A. Friedman, Helen Remotti, Tito Fojo, Hee Won Yang, Gavin Thurston, Minah Kim

**Affiliations:** 1 Department of Pathology and Cell Biology,; 2 Department of Biomedical Informatics, and; 3 Department of Medicine, Columbia University Irving Medical Center, New York, New York, USA.; 4 Regeneron Pharmaceuticals Inc., Tarrytown, New York, USA.

**Keywords:** Angiogenesis, T cells

## Abstract

Improving the management of metastasis in pancreatic neuroendocrine tumors (PanNETs) is critical, as nearly half of patients with PanNETs present with liver metastases, and this accounts for the majority of patient mortality. We identified angiopoietin-2 (ANGPT2) as one of the most upregulated angiogenic factors in RNA-Seq data from human PanNET liver metastases and found that higher ANGPT2 expression correlated with poor survival rates. Immunohistochemical staining revealed that ANGPT2 was localized to the endothelial cells of blood vessels in PanNET liver metastases. We observed an association between the upregulation of endothelial ANGPT2 and liver metastatic progression in both patients and transgenic mouse models of PanNETs. In human and mouse PanNET liver metastases, ANGPT2 upregulation coincided with poor T cell infiltration, indicative of an immunosuppressive tumor microenvironment. Notably, both pharmacologic inhibition and genetic deletion of ANGPT2 in PanNET mouse models slowed the growth of PanNET liver metastases. Furthermore, pharmacologic inhibition of ANGPT2 promoted T cell infiltration and activation in liver metastases, improving the survival of mice with metastatic PanNETs. These changes were accompanied by reduced plasma leakage and improved vascular integrity in metastases. Together, these findings suggest that ANGPT2 blockade may be an effective strategy for promoting T cell infiltration and immunostimulatory reprogramming to reduce the growth of liver metastases in PanNETs.

## Graphical abstract

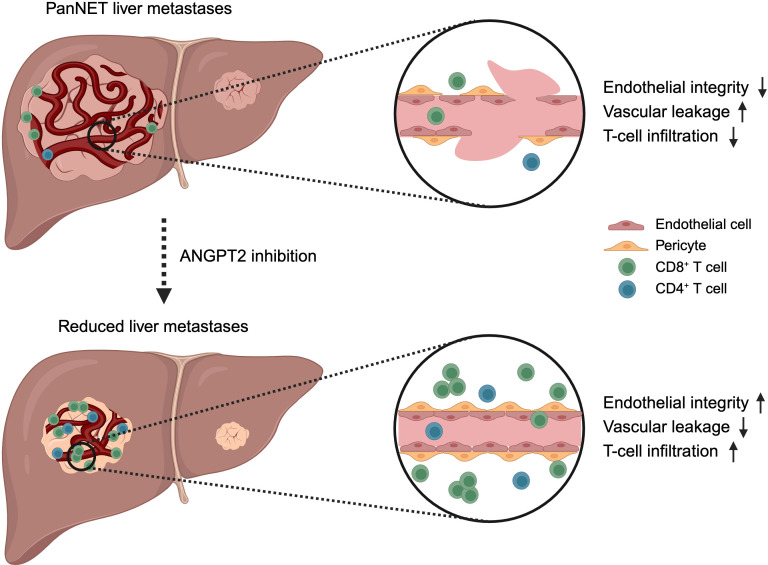

## Introduction

Pancreatic neuroendocrine tumors (PanNETs) account for approximately 3%–5% of all pancreatic cancers. Their incidence has been steadily increasing over recent decades; however, they remain an understudied area of pancreatic cancer (1–3). Nearly half of all newly diagnosed patients with PanNETs present with liver metastasis, which correlates with poor prognosis, and about 50% of patients who experience tumor recurrence after surgical resection of the primary tumor develop liver metastasis (4–6). Furthermore, about half of patients with various malignancies either present with or develop liver metastasis (7, 8), emphasizing an urgent and unmet need to understand the mechanisms underlying the progression of liver metastasis.

Cancer immunotherapy, which stimulates antitumor immunity, has shown remarkable efficacy in the treatment of many aggressive malignancies (9–11). Despite substantial advances in immunotherapy, such as immune checkpoint inhibitor therapy, recent clinical trials of immune checkpoint inhibitor monotherapy in patients with advanced PanNETs had a less than 10% objective response rate (12, 13). This limited response rate to immunotherapy is largely due to the immunosuppressive nature of the tumor microenvironment, which is exacerbated by poor immune cell infiltration. Consistent with other solid tumors, extensive lymphocyte infiltration of primary neuroendocrine tumors is associated with improved median recurrence-free survival (14). Although metastasis is the primary cause of cancer mortality, the signals that drive T cell infiltration into metastases remain largely unknown.

Tumor blood vessels are structurally and functionally abnormal and unstable and undergo sprouting angiogenesis (15). Recent clinical and preclinical studies have shown that the efficacy of immune checkpoint inhibitors for cancer therapy can be enhanced by blocking VEGFA (16, 17). This potentiation is thought to result from improved vascular function (18), which can promote activation and perivascular accumulation of T cells (19, 20).

PanNETs are characterized by extreme vascularity, consistent with the beneficial effects of angiogenesis inhibitors in patients with PanNETs. Antiangiogenic drugs such as sunitinib and bevacizumab that target VEGF signaling have shown efficacy in patients with well-differentiated, metastatic, or nonresectable PanNETs (21–24). However, the therapeutic success of anti-VEGF agents in patients with advanced PanNETs is limited by postoperative recurrence and liver metastasis in about 60% of patients treated with sunitinib (25). Moreover, preclinical studies showed that prolonged inhibition of VEGF signaling can paradoxically fuel tumor progression and metastatic spread after an initial favorable response (26, 27). Angiopoietin-2 (ANGPT2), which binds to the receptor tyrosine kinase TIE2 in endothelial cells, is a cooperative driver of angiogenesis and vascular destabilization (28). Increased ANGPT2 expression often correlates with poor prognosis and disease progression in many types of solid tumors (29–31). Unlike another member of the angiopoietin family, angiopoietin-1 (ANGPT1), which constitutively induces TIE2 activation and promotes vascular maturation and stabilization, ANGPT2 functions as a context-dependent agonist/antagonist (32–34). Consistent with other studies, our previous work has shown that ANGPT2 can suppress TIE2 signaling, thereby promoting vascular destabilization and leakage (35). However, the contribution of ANGPT2-mediated vascular destabilization to tumor immune escape in metastatic disease remains unclear.

In this study, we investigated the mechanism through which vascular destabilization contributes to PanNET liver metastases and disease progression. We identified ANGPT2 as one of the most upregulated angiogenic factors in the RNA-Seq data from human PanNET liver metastases and found that its increased expression correlated with poor overall survival. Building on this, we determined whether ANGPT2 blockade could slow the growth of liver metastases in PanNET mouse models. Pharmacologic blockade or genetic deletion of ANGPT2 suppressed liver metastasis and extended survival in mice with metastatic PanNETs. Both human and mouse PanNET liver metastases showed a “cold” immune microenvironment, characterized by minimal T cell infiltration, which was associated with ANGPT2 upregulation. To understand the mechanisms underlying the antimetastatic action of ANGPT2 blockade, we investigated whether ANGPT2 inhibition had effects on vascular changes and T cell infiltration and activation in liver metastases. Our experiments revealed that ANGPT2 blockade enhanced vascular integrity, reduced vascular leakage, and promoted T cell infiltration with an increase in activated T cells in the context of liver metastases. Taken together, our data provide further evidence that ANGPT2 blockade promotes immunostimulatory reprogramming and reduces the progression of PanNETs with liver metastasis.

## Results

### Endothelial ANGPT2 increases with liver metastasis in patients with PanNETs and PanNET mice.

**Figure 1:**
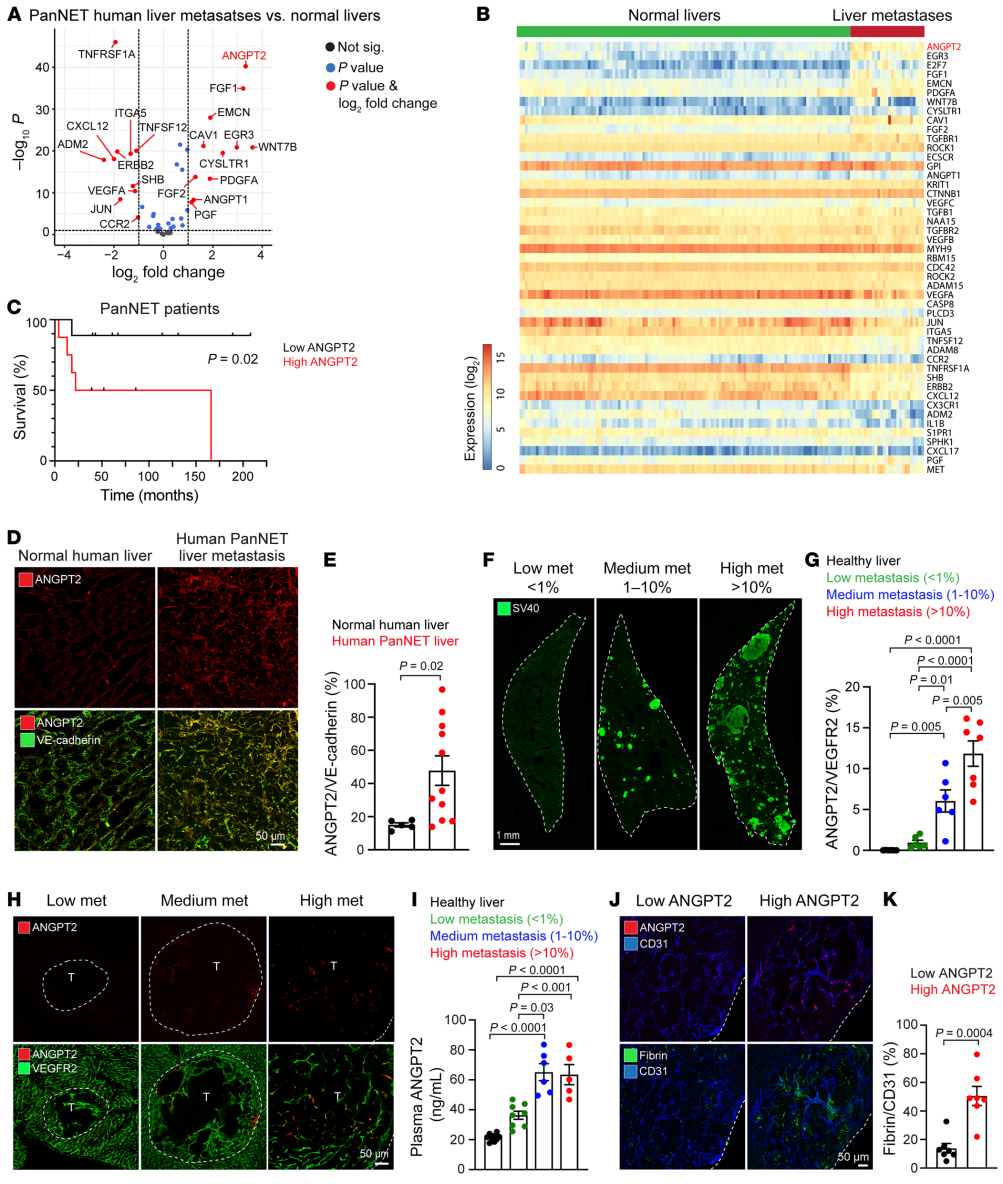
Increased ANGPT2 expression in PanNET liver metastases. (**A** and **B**) Volcano plot (**A**) and heatmap (**B**) from RNA-Seq transcriptome analyses of differentially expressed angiogenesis-related genes in liver metastases of patients with PanNETs (*n* = 30) and normal human liver tissues (*n* = 119), with ANGPT2 noted in red. (**A**) The horizontal dotted line in the volcano plot represents a FDR of 1%; the vertical lines represent the threshold (±2-fold) of the log_2_ fold change. (**B**) The heatmap shows normalized RNA-Seq data for angiogenesis-related genes (rows) from 149 samples (columns). (**C**) Kaplan-Meier survival curves of patients with PanNETs with low (*n* = 9) or high (*n* = 11) concentrations of plasma ANGPT2 (cutoff, 0.66 ng/mL). (**D** and **E**) Increased ANGPT2 immunoreactivity in liver metastases of patients with PanNETs (*n* = 11) compared with that in normal livers (*n* = 5) (scale bar: 50 μm) (**D**) and quantification of ANGPT2^+^ vessels (unpaired *t* test) (**E**). (**F**–**H**) Whole-liver-lobe cross-sections showing the metastatic tumor progression of RT2;AB6F1 mice (scale bar: 1 mm) (**F**). Mice at 15, 18, and 20 weeks of age were stratified by percentage area of metastasis (low, <1%; medium, 1%–10%; high, >10%). Quantification of ANGPT2^+^ vessels (1-way ANOVA with Tukey’s multiple comparisons test) (**G**) and representative images showing increased ANGPT2 during metastatic growth (scale bar: 50 μm) (**H**). (**I**) Analysis of plasma ANGPT2 concentrations by ELISA (1-way ANOVA with Tukey’s multiple comparisons test). (**J** and **K**) Greater vascular leakage marked by extravasated fibrin in metastatic colonies of the liver with high ANGPT2 compared with colonies with little or no ANGPT2 staining in RT2;AB6F1 mice at 20 weeks of age (scale bar: 50 μm) (**J**) and its quantification (unpaired *t* test) (**K**). The cutoff value for high and low ANGPT2 was 13.8, the average ANGPT2 expression (ANGPT2/CD31, %). For **E**, **G**, **I**, and **K**, each data point represents an individual human or mouse. Data are displayed as the mean ± SEM.

To identify potential vascular regulators involved in PanNET metastases, we conducted an RNA-Seq analysis focusing on angiogenesis-related genes, using an established RNA-Seq data set of 30 human PanNET liver metastases (36) and 119 healthy livers obtained from the Genotype-Tissue Expression (GTEx) biobank. Our comparative transcriptomics identified *ANGPT2* as a differentially expressed gene in human PanNET liver metastases, with a 10.5-fold increase compared with healthy liver samples (Figure 1, A and B). Additionally, we observed an elevated level of *ANGPT2* in human PanNET primary tumor samples as compared with healthy pancreatic samples in the RNA-Seq data set. Differential expression of some angiogenic factors, such as *PDGFA,* was also noted when comparing human primary PanNETs to liver metastases (Supplemental Figure 1, A and B; supplemental material available online with this article; https://doi.org/10.1172/JCI167994DS1). Furthermore, patients with higher concentrations of circulating ANGPT2 in plasma (cutoff, 0.66 ng/mL, average ANGPT2 concentration) showed poor survival compared with patients with PanNETs with lower levels of ANGPT2 (Figure 1C and Supplemental Figure 2, A and B). In line with the transcriptomic analyses, ANGPT2 immunoreactivity was stronger in the vessels of liver metastases from patients with PanNETs than in those of adjacent normal liver (Figure 1, D and E).

C57BL/6 RIP-Tag2 B6 (RT2;B6) mice have a low frequency of liver metastasis and high mortality after 12 weeks due to hypoglycemia from insulin-producing PanNETs. To address the limitations observed in RT2;B6 mice, we created RT2;AB6F1 mouse hybrids, bred from female animals with an A/J genetic background and RT2;B6 male animals, based on previous studies (37, 38). We confirmed that RT2;AB6F1 hybrids had extensive liver metastases but longer survival due to less severe hypoglycemia (Supplemental Figure 3, A–D).

Using the RT2;AB6F1 mouse model to recapitulate the poorly functional, highly metastatic clinical PanNETs, we classified mice aged 15–20 weeks into 3 groups based on the metastatic burden (percentage SV40^+^ area) in the liver: low (<1%), medium (1%–10%), or high (>10%) fractional area of liver (Figure 1F). Examination of ANGPT2 immunoreactivity in blood vessels of normal liver and PanNET metastases revealed little or no immunoreactivity in normal sinusoids but clear immunostaining in metastases that increased with metastatic growth (Figure 1, G and H), which was also consistent with increased ANGPT2 levels in plasma (Figure 1I). We also observed a progressive increase in the expression of other angiogenic genes — *VEGFA*, *HIF1A*, *PDGFA*, and *FGF1* — accompanying metastatic growth. However, there were no notable changes in the expression of other genes related to the angiopoietin-TIE pathway — *ANGPT1*, *TIE1*, and *TIE2* (Supplemental Figure 1C). Higher ANGPT2 expression in liver metastases was accompanied by greater vascular leakage, as evidenced by perivascular fibrin staining (Figure 1, J and K). Taken together, these data show a strong association of endothelial ANGPT2 with PanNET metastatic burden in the liver.

### ANGPT2 inhibition suppresses liver metastasis and prolongs survival in PanNETs.

**Figure 2:**
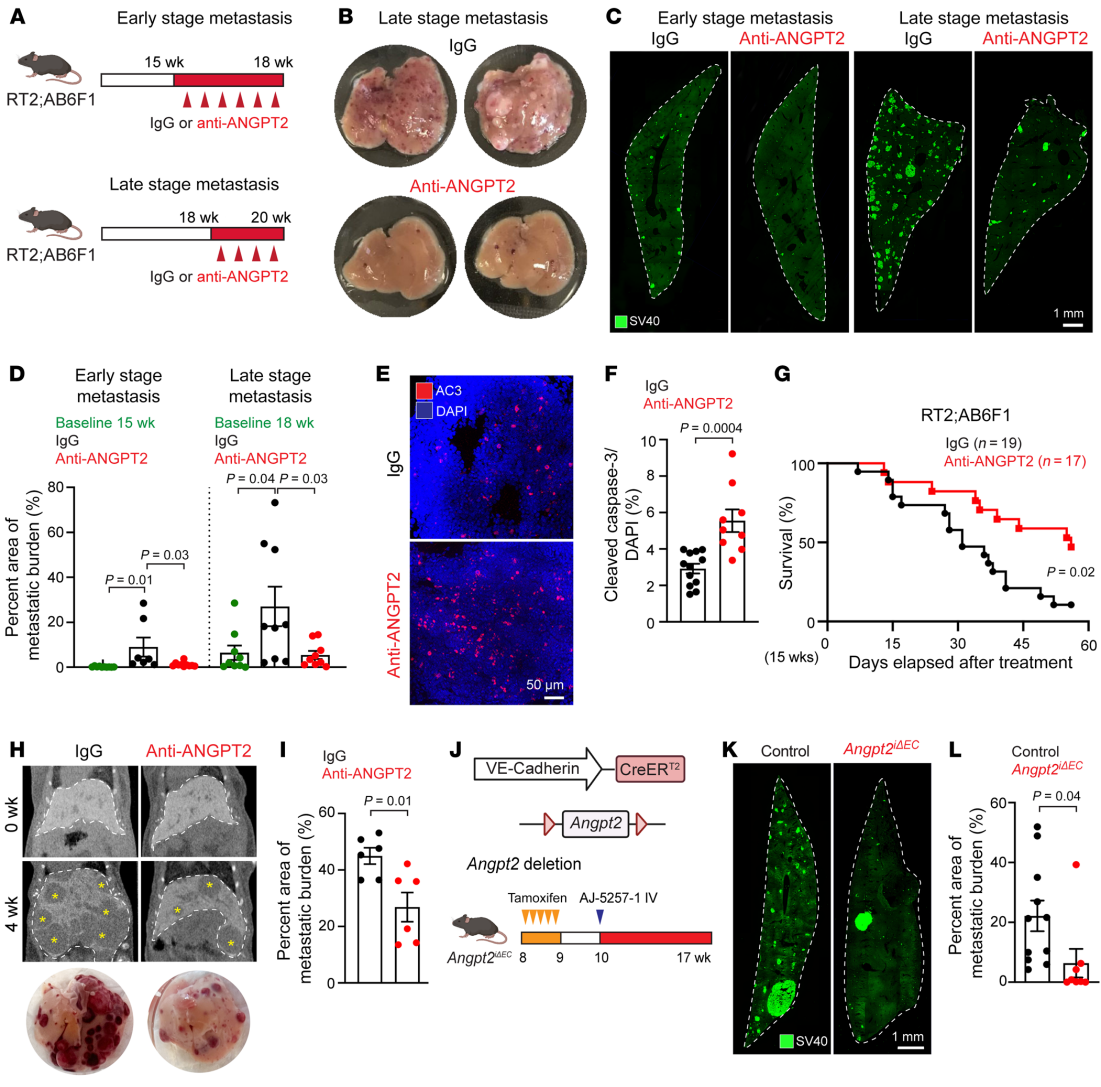
ANGPT2 inhibition suppresses liver metastases and improves overall survival in mice with metastatic PanNETs. (**A**) Experimental timeline for treatments in RT2;AB6F1 mice beginning at 15 (early-stage metastasis) or 18 weeks (late-stage metastasis) of age, followed by perfusion at 18 or 20 weeks of age, respectively. (**B**) Gross view images of liver lobes from RT2;AB6F1 mice treated with IgG or anti-ANGPT2 at 20 weeks of age. (**C** and **D**) Metastatic burden of whole-liver-lobe cross-sections in RT2;AB6F1 mice treated with IgG or anti-ANGPT2 starting at 15 or 18 weeks of age (scale bar: 1 mm) (**C**), and corresponding quantification of SV40 T-antigen^+^ metastatic burden (**D**). Quantification in **D** compares liver metastatic burden at the onset of treatment and 15 or 18 weeks (1-way ANOVA with Tukey’s multiple comparisons test). (**E** and **F**) Representative images comparing apoptosis, measured by cleaved caspase-3 in liver metastases of 20-week-old RT2;AB6F1 mice treated with IgG or anti-ANGPT2 (scale bar: 50 μm) (**E**) and its quantification (unpaired *t* test) (**F**). (**G**) Kaplan-Meier survival curves of RT2;AB6F1 mice treated with IgG or anti-ANGPT2 (IgG, *n* = 19; anti-ANGPT2, *n* = 17). (**H** and **I**) Micro-CT images (top) and macroscopic images of livers (bottom) showing decreased liver metastases in AJ-5257-1–injected mice treated with anti-ANGPT2 compared with those treated with IgG. Asterisks indicate individual metastatic lesions in the liver. (**H**) Quantification of SV40 T-antigen^+^ metastatic burden (unpaired *t* test) (**I**). (**J**) Schematic of inducible Cre-*loxP* system in *Angpt2^iΔEC^* mice for endothelial *Angpt2* depletion (top). Experimental timeline for *Angpt2^iΔEC^* mice treated with tamoxifen and, subsequently, inoculated with AJ-5257-1 cells after 7 weeks (bottom). (**K** and **L**) Reduced metastatic burden in *Angpt2^iΔEC^* mice compared with that in control mice (scale bar: 1 mm) (**K**) and corresponding quantification of SV40 T-antigen^+^ metastatic burden (unpaired *t* test) (**L**). For **D**, **F**, **I**, and **L**, each data point indicates an individual mouse. Data are displayed as the mean ± SEM.

To investigate the antimetastatic effects of ANGPT2 blockade, we conducted a series of experiments targeting ANGPT2 in metastatic PanNET mouse models. For pharmacologic ANGPT2 inhibition, we used an anti-ANGPT2 antibody (REGN910) to treat RT2;AB6F1 mice from 15 to 18 weeks of age (early-stage metastasis) and from 18 to 20 weeks of age (late-stage metastasis) (Figure 2A). Remarkably, inhibition of ANGPT2 delayed growth of liver metastases at treatment onset in both the early- and late-stage groups (Figure 2, B–D). Apoptotic tumor cells, marked by cleaved caspase-3, were greater in metastases of mice treated with anti-ANGPT2 than in IgG-treated controls (Figure 2, E and F, and Supplemental Figure 4, A and B). ANGPT2 blockade also reduced the burden of primary PanNETs in RT2;AB6F1 mice when the treatment started at 18 weeks of age but not at 15 weeks of age (Supplemental Figure 5, A and B). This age-dependent difference in the efficacy of ANGPT2 blockade in primary tumors may be attributed to temporal changes in the expression of ANGPT2. Specifically, 15-week primary tumors showed significantly lower ANGPT2 immunoreactivity compared with 18-week primary tumors (Supplemental Figure 5, C and D). Furthermore, prolonged treatment of RT2;AB6F1 mice with the anti-ANGPT2 antibody from 15 to 23 weeks of age significantly improved survival (Figure 2G). To exclude possible complications associated with primary tumors, we tested the effects of ANGPT2 inhibition in wild-type AJ mice following tail vein injection of AJ-5257-1 cells, which demonstrated a preferential metastasis to the liver compared with the lungs (38). Consistent with the results in RT2;AB6F1 mice, treatment with anti-ANGPT2 antibody reduced the growth of liver metastases in mice injected with AJ-5257-1 cells (Figure 2, H and I). Micro-CT imaging analysis showed that ANGPT2 blockade delayed growth of liver metastases and reduced metastatic burden over a 4-week treatment period in mice injected with AJ-5257-1 cells (Figure 2H and Supplemental Figure 6).

We generated *Angpt2^iΔEC^* mice, which have an endothelial cell–specific deletion of ANGPT2, to complement the pharmacologic approach to ANGPT2 blockade. These mice were produced by crossing *Angpt2^fl/fl^* mice (39, 40) with VE-cadherin–Cre-ER^T2^ mice (41), and they were injected with AJ-5257-1 cells intravenously (Figure 2J). *Angpt2^iΔEC^* mice developed liver metastases with little or no ANGPT2 immunoreactivity on blood vessels (Supplemental Figure 7). Compared with wild-type mice, liver metastases in *Angpt2^iΔEC^* mice were significantly smaller (Figure 2, K and L). These results provide evidence that ANGPT2 blockade or deletion reduces liver metastases and improves survival in mice with metastatic PanNETs.

### ANGPT2 inhibition enhances vascular integrity in PanNET liver metastases.

**Figure 3:**
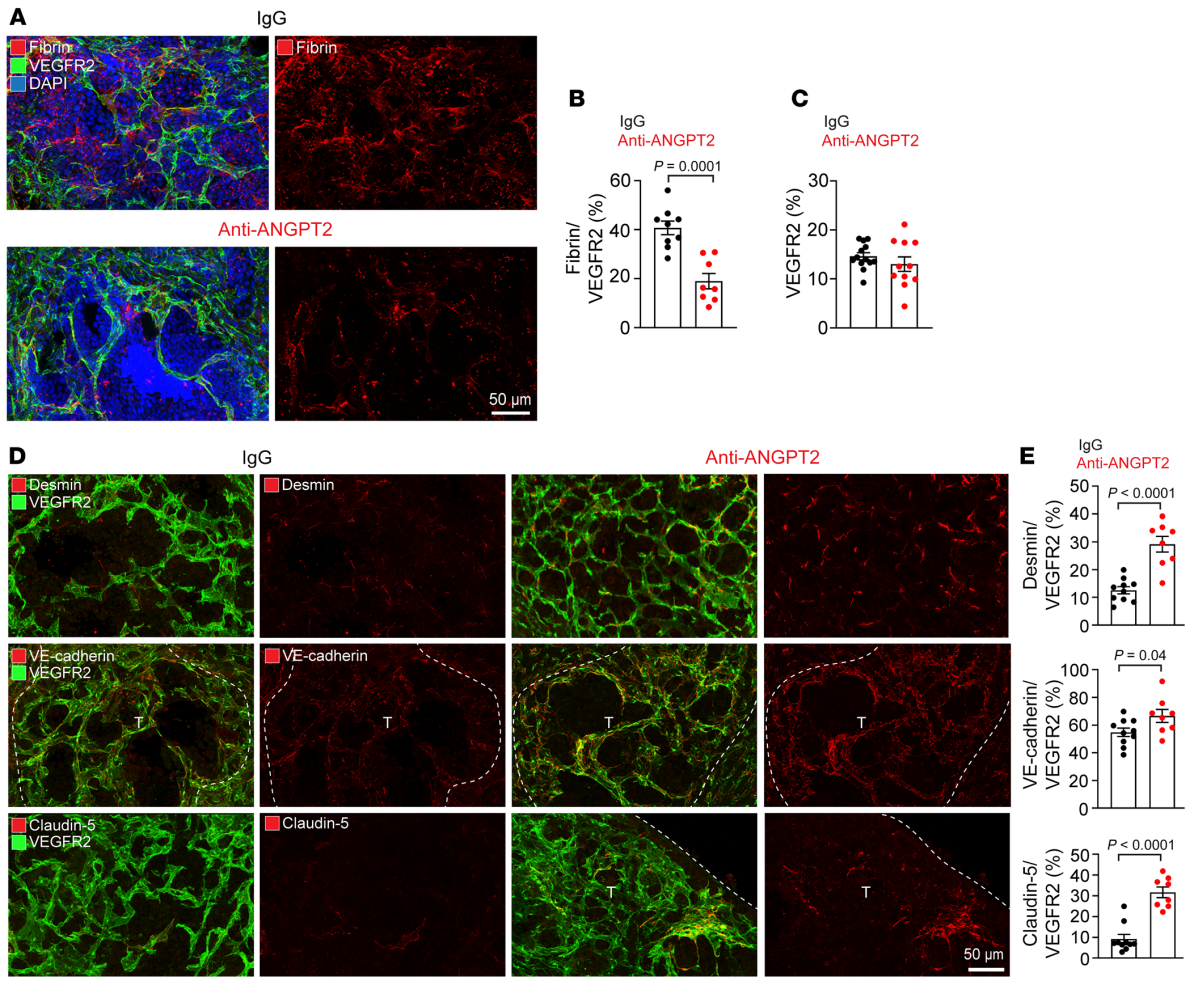
ANGPT2 inhibition restores vessel integrity in PanNET liver metastases. In RT2;AB6F1 mice, anti-ANGPT2 and IgG were administered twice per week starting at 18 weeks. Mice were perfused after 2 weeks of treatment at 20 weeks of age. (**A** and **B**) Alleviation of vascular leakage, as evidenced by decreased extravasation of fibrin in metastatic colonies, in mice treated with anti-ANGPT2 (scale bar: 50 μm) (**A**) and its quantification (unpaired *t* test) (**B**). (**C**) Comparison of VEGFR2^+^ vascular density in liver metastases of RT2;AB6F1 mice treated with IgG or anti-ANGPT2 (unpaired *t* test). (**D** and **E**) Representative images comparing desmin^+^ pericyte coverage and VE-cadherin^+^ and claudin-5^+^ endothelial cell junctions in metastatic regions from 20-week-old RT2;AB6F1 mice treated with IgG or anti-ANGPT2 (scale bar: 50 μm) (**D**), and the corresponding quantification of desmin^+^, VE-cadherin^+^, and claudin-5^+^ sinusoids (unpaired *t* test) (**E**). Dotted lines in **D** indicate metastatic tumor regions indicated by “T.” For **B**, **C**, and **E**, each data point represents an individual mouse. Data are displayed as the mean ± SEM.

Improved vascular function in tumors treated with antiangiogenic agents is accompanied by reprogramming of the immune microenvironment and increased antitumor immunity (19, 42). To elucidate the mechanisms responsible for suppressing liver metastatic growth following ANGPT2 blockade, we investigated the potential of ANGPT2 inhibition to improve vascular integrity in PanNET liver metastases. In RT2;AB6F1 mice, liver metastases showed a reduction in perivascular fibrin when mice were treated with the anti-ANGPT2 antibody between the ages of 18 to 20 weeks (Figure 3, A and B). Additionally, the treatment decreased fibrin extravasation in primary tumors at 20 weeks, when the antitumor effect was observed, but not at 18 weeks (Supplemental Figure 5, E and F). While the vascular density in the metastases remained relatively unchanged after treatment with anti-ANGPT2 or IgG (Figure 3C), blood vessels in the metastases exhibited several changes after anti-ANGPT2 treatment. Notably, there was a 2.3-fold increase in desmin^+^ pericyte coverage and enhanced staining for VE-cadherin at adherens junctions by 1.2-fold and claudin-5 at tight junctions by 3.4-fold (Figure 3, D and E). Taken together, our findings suggest that ANGPT2 blockade may mitigate vascular leakage and enhance vascular integrity in PanNET liver metastases.

### ANGPT2 inhibition improves T cell infiltration of PanNET liver metastases.

**Figure 4:**
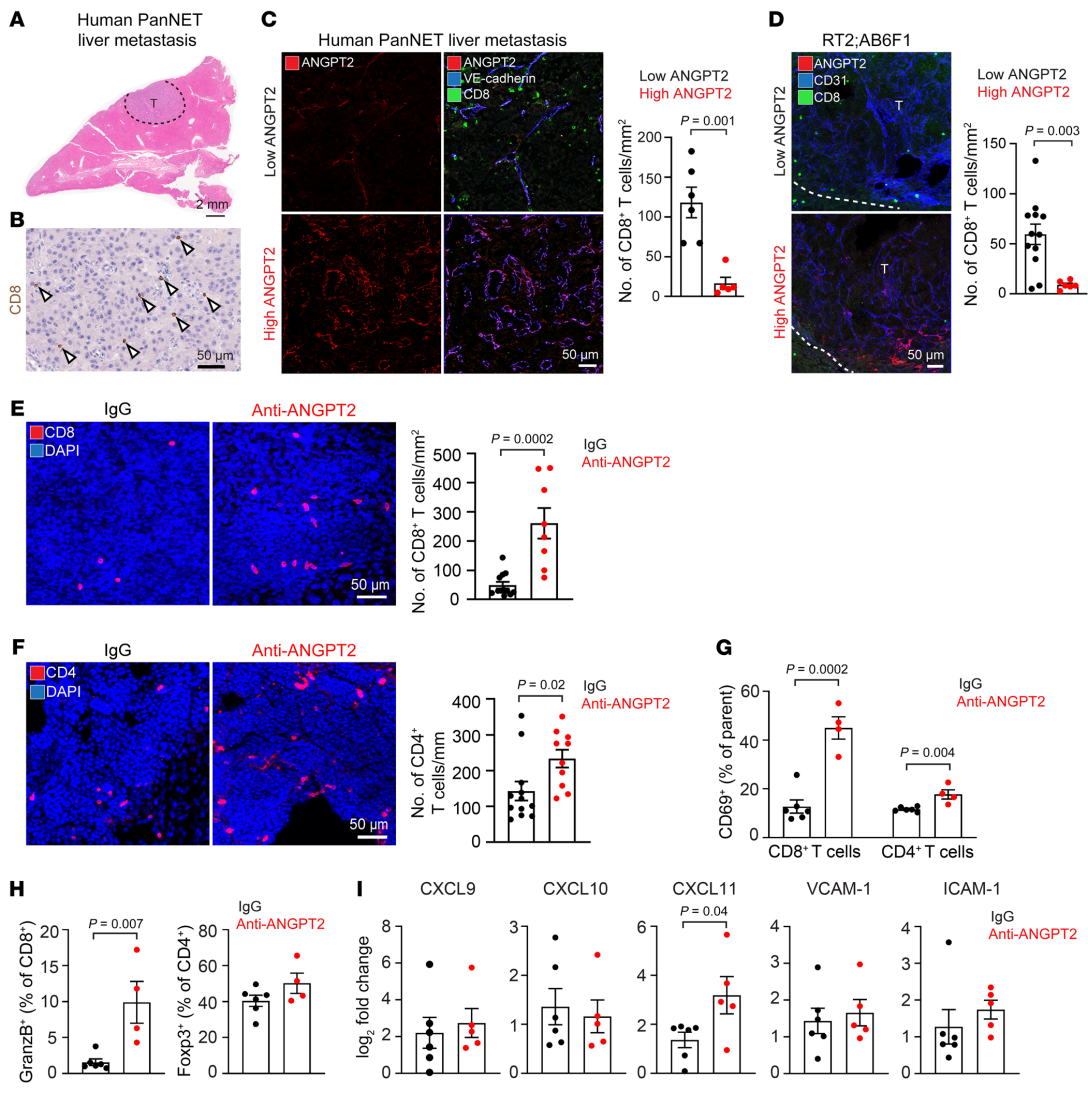
ANGPT2 regulates T cell infiltration in PanNET liver metastases. (**A**) Human PanNET liver metastasis (T) stained by H&E, outlined with dashes (scale bar: 2 mm). (**B**) CD8^+^ T cells in human PanNET liver metastasis. Arrowheads mark individual CD8^+^ T cells (scale bar: 50 μm). (**C**) CD8^+^ T cell infiltration into the low ANGPT2-expressing (top) or high ANGPT2-expressing (bottom) metastatic livers from patients with PanNETs and corresponding quantification (unpaired *t* test) (scale bar: 50 μm). The cutoff value for high and low ANGPT2 was 13.8, the average ANGPT2 coverage (ANGPT2/CD31, %). (**D**) Analysis of CD8^+^ T cell infiltration into the liver metastases with low ANGPT2 (top) or high ANGPT2 (bottom) expressions from 18- to 20-week-old RT2;AB6F1 mice and corresponding quantification (unpaired *t* test) (scale bar: 50 μm). The cutoff value for high and low ANGPT2 was 13.4, average ANGPT2 expression (ANGPT2/CD31, %). (**E** and **F**) Increased CD8^+^ (**E**) and CD4^+^ (**F**) T cell infiltration into the liver metastases in 20-week-old RT2;AB6F1 mice treated with anti-ANGPT2 for 2 weeks compared with low T cell infiltration in IgG-treated control mice and corresponding quantifications (unpaired *t* test) (scale bar: 50 μm). (**G** and **H**) Analysis of flow cytometry showing frequencies of activated (CD69^+^) CD8^+^ or CD4^+^ T cells (**G**), activated (granzyme B^+^) CD8^+^ T cells (**H**, left) and regulatory (Foxp3^+^) CD4^+^ T cells (**H**, right) in the experimental metastasis mouse model inoculated with AJ-5257-1 cells (unpaired *t* test). Three weeks after inoculation, mice were treated with IgG or anti-ANGPT2 twice per week for 4 weeks and perfused 7 weeks after inoculation. (**I**) Analysis of gene expression patterns for CXCL9, CXCL10, CXCL11, VCAM-1, and ICAM-1 by qPCR using the metastatic liver tissues of 20-week-old RT2;AB6F1 mice treated with either IgG or anti-ANGPT2 (unpaired *t* test). For **C**–**I**, each data point represents an individual human or mouse. Data are displayed as the mean ± SEM.

The infiltration of immune cells into the tumor microenvironment is essential for promoting an antitumor immune response and is necessary for the success of immunotherapies (43). Consistent with previous evidence of a cold tumor microenvironment in PanNETs (44, 45), our findings showed that human PanNET liver metastases had low numbers of both CD8^+^ (Figure 4, A and B) and CD4^+^ T cells (data not shown).

To understand the correlation between ANGPT2 and CD8^+^ T cell infiltration of human PanNET liver metastases, we categorized specimens based on their degree of endothelial ANGPT2 immunoreactivity. Metastases with strong ANGPT2 staining had significantly fewer CD8^+^ T cells than those with weak ANGPT2 staining in patients (Figure 4C). Similarly, in RT2;AB6F1 mice, liver metastases exhibiting a higher ANGPT2 immunoreactivity in blood vessels were associated with a reduced presence of CD8^+^ T cells (Figure 4D). A similar pattern was observed for CD4^+^ T cells in both human and mouse PanNET liver metastases (Supplemental Figure 8, A and B).

Next, to determine whether ANGPT2 can suppress T cell infiltration of metastases, we measured CD8^+^ and CD4^+^ T cells in liver metastases of RT2;AB6F1 mice with or without pharmacologic ANGPT2 inhibition. Notably, after ANGPT2 blockade, the density of CD8^+^ and CD4^+^ T cells in metastases was significantly greater than in IgG-treated controls (Figure 4, E and F). In the primary tumors treated with IgG, we observed a higher density of CD8^+^ T cells within the tumor periphery than in the tumor core. While anti-ANGPT2 treatment did not alter the density of CD8^+^ T cells in either the tumor periphery or the tumor core at 18 weeks, it significantly enhanced the infiltration of CD8^+^ T cells into both these areas by 20 weeks (Supplemental Figure 9, A and B).

Building on our findings that ANGPT2 inhibition can enhance T cell infiltration into both primary and metastatic PanNETs, we investigated the potential therapeutic efficacy of combining anti-ANGPT2 with an anti–programmed cell death protein-1 (anti–PD-1) checkpoint inhibitor (Supplemental Figure 10A). As expected, due to the immunosuppressive tumor microenvironment of PanNET, we found resistance to anti–PD-1 monotherapy, indicated by a liver metastatic burden similar to that of control mice (Supplemental Figure 10, B and C). However, the metastatic burden significantly decreased when anti-PD-1 was combined with anti-ANGPT2, mirroring the effect of anti-ANGPT2 monotherapy. This implies no additive or enhanced benefit from combining anti-ANGPT2 and anti–PD-1 for the treatment of liver metastases in PanNET.

Further immune analysis using flow cytometry showed that ANGPT2 inhibition in mice injected with AJ-5257-1 cells increased the proportion of activated (CD69^+^ or granzyme B^+^) CD8^+^ T cells in liver metastases but not the effector memory (CD44^hi^ CD62L^lo^) CD8^+^ T cells (Figure 4, G and H, and Supplemental Figure 8C). The population of activated (CD69^+^) CD4^+^ T cells was also increased with no changes in effector memory (CD44^hi^ CD62L^lo^) or regulatory (Foxp3^+^) CD4^+^ T cells in the liver metastases following ANGPT2 inhibition (Figure 4, G and H, and Supplemental Figure 8C). We also explored changes in the density of myeloid cells in the liver metastases of RT2;AB6F1 mice after ANGPT2 inhibition. The ANGPT2 blockade resulted in a decreased proportion of TIE2-expressing monocytes, despite their total frequency being low (0.15% of CD45^+^ cells). There were no significant changes in the frequencies of M1 macrophages (CD206^lo^CD11c^hi^), dendritic cells (MHC II^+^CD11c^+^), and monocytic myeloid-derived suppressor cells (CD11b^+^Ly6C^+^Ly6G^–^). However, there was an approximate 3-fold reduction in M2 macrophages (CD206^hi^CD11c^lo^) in liver metastases following ANGPT2 inhibition (Supplemental Figure 8, D–G).

To better understand the mechanism by which vascular changes resulting from ANGPT2 inhibition promote T cell infiltration in liver metastases, we examined the expression patterns of chemokines and adhesion molecules crucial for T cell recruitment (46) using quantitative PCR (qPCR). Our study revealed a significant increase in the expression of CXCL11, but not CXCL9 and CXCL10, after ANGPT2 blockade in the liver metastases of RT2;AB6F1 mice (Figure 4I). Furthermore, we observed no significant differences in the expression of adhesion molecules, including vascular cell adhesion molecule 1 (VCAM-1) and intracellular adhesion molecule 1 (ICAM-1) (Figure 4I). Collectively, our results suggest that ANGPT2 exerts a potentially immunosuppressive effect on T cell immunity.

### ANGPT2 blockade reduces growth of PanNET liver metastases by increasing T cell influx.

**Figure 5:**
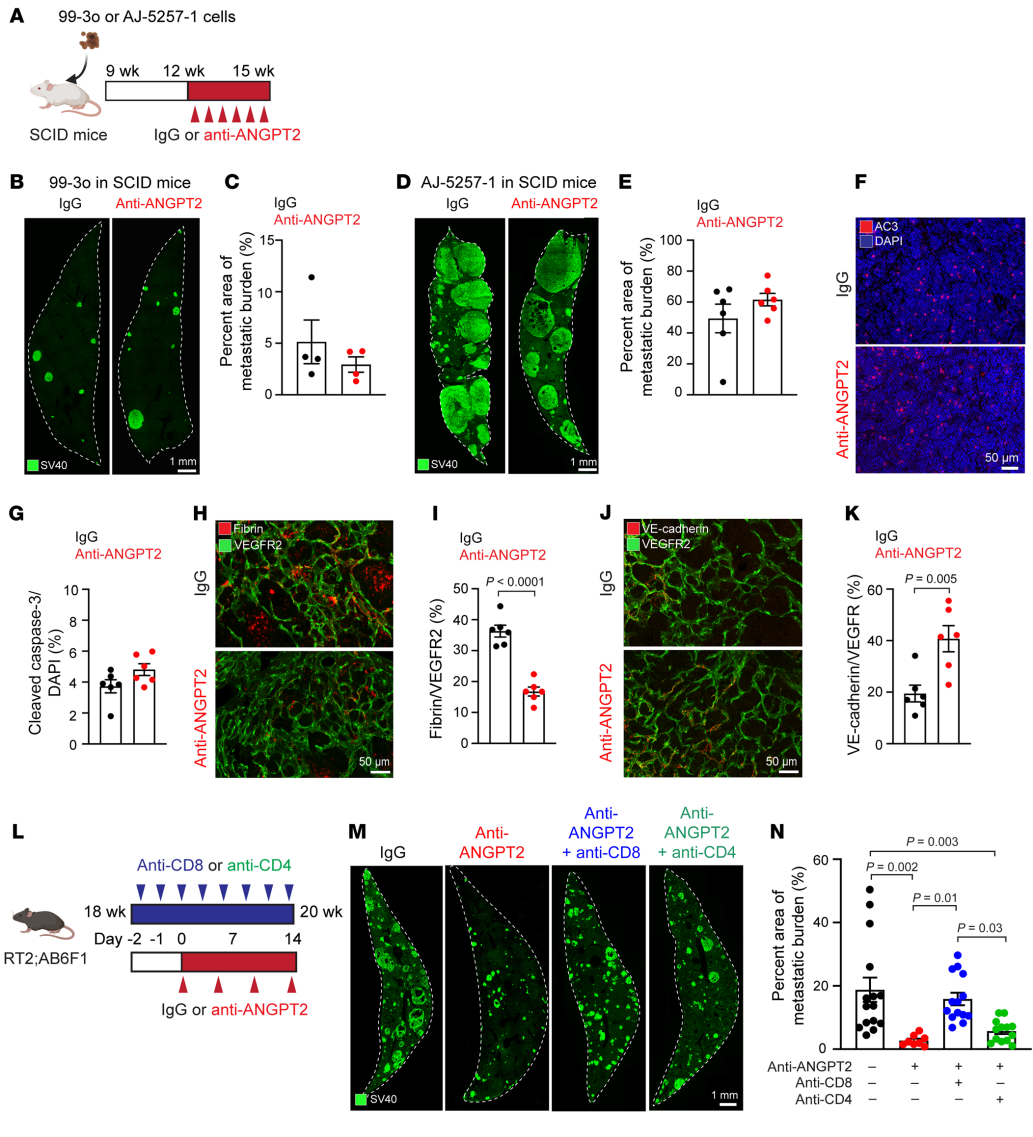
T cells mediate the antimetastatic response of ANGPT2 blockade. (**A**) Experimental timeline for SCID mice injected with 99-3o or AJ-5257-1 cells. (**B**–**E**) Whole-liver-lobe cross-sections comparing metastatic burden in 99-3o cell–injected (scale bar: 1 mm) (**B**) or AJ-5257-1 cell–injected (scale bar: 1 mm) (**D**) SCID mice treated with IgG or anti-ANGPT2, and the corresponding quantifications of SV40 T-antigen^+^ metastatic burden (unpaired *t* test) (**C** and **E**). (**F** and **G**) Expression of cleaved caspase-3 in 20-week-old RT2;AB6F1 mice treated with IgG (top) or anti-ANGPT2 (bottom) (scale bar: 50 μm) (**F**) and its quantification (unpaired *t* test) (**G**). (**H**–**K**) Expression of fibrin (scale bar: 50 μm) (**H**) and VE-cadherin (scale bar: 50 μm) (**J**) in 20-week-old RT2;AB6F1 mice treated with IgG or anti-ANGPT2 and quantification of vascular leakage (**I**) and VE-cadherin coverage (**K**) (unpaired *t* test). (**L**) Experimental timeline of CD8^+^ or CD4^+^ T cell depletion study in which RT2;AB6F1 mice were treated with IgG, anti-ANGPT2, and combination of anti-ANGPT2 with anti-CD8 or anti-CD4 antibodies beginning at 18 weeks of age. (**M** and **N**) Whole-liver-lobe cross-sections comparing metastatic burden in RT2;AB6F1 mice treated with IgG, anti-ANGPT2, and combination of anti-ANGPT2 with anti-CD8 or anti-CD4 antibodies (scale bar: 1 mm) (**M**) and quantification of SV40 T-antigen^+^ metastatic burden (1-way ANOVA with Tukey’s multiple comparisons test) (**N**). For **C**, **E**, **G**, **I**, **K**, and **N**, each data point indicates an individual mouse. Data are displayed as mean ± SEM.

To test the hypothesis that ANGPT2 blockade can reduce the growth of PanNET liver metastases by augmenting T cell infiltration, we investigated whether the antimetastatic effects of ANGPT2 inhibition were negated in SCID mice, which lack both T and B cells. We injected 2 distinct mouse-derived PanNET cell lines, 99-3o and AJ-5257-1, into SCID mice for a comparative study (Figure 5A). We selected these cell lines based on their differing metastatic capabilities when tested in RT2;B6 mice, with AJ-5257-1 cells being more aggressive than 99-3o cells (38). We found that the anti-ANGPT2 antibody did not slow the growth of liver metastases in SCID mice (Figure 5, B–E). The degree of apoptosis in liver metastases in SCID mice remained consistent after treatment with either the anti-ANGPT2 antibody or the control IgG (Figure 5, F and G). Despite the limited reduction in metastatic burden in SCID mice treated with anti-ANGPT2, liver metastatic regions had less extravasated fibrin and stronger VE-cadherin staining at endothelial cell junctions than IgG controls, mirroring our findings in immunocompetent mice (Figure 5, H–K).

To confirm the contribution of T cells to the antimetastatic effect of ANGPT2 blockade in the liver, we depleted CD8^+^ or CD4^+^ T cells by administration of anti-CD8 or anti-CD4 antibodies 2 days before introducing the anti-ANGPT2 antibody. This treatment persisted for 2 weeks, after which we assessed the metastatic burden in the livers of 20-week-old RT2;AB6F1 mice (Figure 5L). Under these conditions, metastatic burden after CD8^+^ T cell depletion plus ANGPT2 inhibition (15.9% metastatic burden) was similar to that in IgG-treated controls, demonstrating an abolishment of the antimetastatic effect of ANGPT2 inhibition (Figure 5, M and N). In contrast, CD4^+^ T cell depletion plus ANGPT2 inhibition (5.7% metastatic burden) did not significantly suppress the antimetastatic effect of ANGPT2 inhibition (2.7% metastatic burden). These findings further emphasize the vital involvement of CD8^+^ T cells and the supplemental role of CD4^+^ T cells in the antimetastatic activity of ANGPT2 blockade in these PanNET liver metastasis models.

## Discussion

This study sought to determine the contribution of endothelial ANGPT2 to the growth of PanNET liver metastases, focusing on vascular changes that influence T cell recruitment. Using human tumor specimens and mouse models of PanNETs, we found multiple lines of evidence supporting the contribution of ANGPT2 to metastatic growth and identifying it as a potential therapeutic target: (a) elevated ANGPT2 levels, both in patient plasma and in RNA transcriptomic analysis of PanNET liver metastases, were associated with a poor prognosis and metastatic progression; (b) in mouse models of PanNETs, the growth of liver metastases was suppressed and survival was prolonged after ANGPT2 inhibition; (c) PanNET liver metastases with high ANGPT2 expression, both in human specimens and mouse models, exhibited sparse T cell infiltration, consistent with a cold immune microenvironment; (d) inhibition of ANGPT2 using a function-blocking antibody increased T cell infiltration and activation while reducing plasma leakage in PanNET liver metastases; and (e) depletion of CD8^+^ T cells negated the antimetastatic effect of the ANGPT2 blocking antibody, while CD4^+^ T cell depletion resulted in only partial suppression. Collectively, the results suggest that ANGPT2 blockade can suppress the growth of PanNET metastases in the liver by improving immunosuppression. These changes are mediated through alterations in tumor blood vessels, promoting the recruitment of CD8^+^ and CD4^+^ T cells.

Given that the majority of cancer mortality results from metastatic disease, it is crucial to understand the mechanisms underlying the vascular regulation of metastatic progression in order to improve patient outcomes. Our experiments were aimed at determining the role of ANGPT2 in the growth of PanNET liver metastases. High mortality of the RT2;B6 model, resulting from hypoglycemia due to insulin-producing PanNET cells (19, 47), has posed challenges for in-depth studies into metastatic progression. To pioneer studies on metastatic PanNETs, we used the RT2;AB6F1 hybrid, which exhibits enhanced metastatic potential to the liver, mirroring the clinical phenotype of advanced disease. To investigate the functional contribution of ANGPT2, we treated mice with PanNET metastases at different stages with an anti-ANGPT2 antibody (REGN910), which is highly specific for ANGPT2. In addition to the genetic deletion of ANGPT2, we used anti-CD8 and anti-CD4 antibodies to assess the contribution of T cells to the antimetastatic effects of ANGPT2 inhibition. Gain-of-function studies could provide additional support to our current findings. Furthermore, considering the heterogeneity of endothelial and immune cells in the liver, as shown by previous single-cell transcriptomic profiling studies (48), analysis of vascular and immune changes at the single-cell level could provide insights into tumor-induced or treatment-induced vascular dynamics and vascular-immune crosstalk in the liver.

Several lines of evidence emphasize the critical role of vascular-immune interactions in mediating antitumor response. Antiangiogenic therapy, such as VEGF blockade, can improve vascular function and facilitate the accumulation of T cells within tumors (18–20). For patients with PanNETs, current antiangiogenic treatments primarily target VEGF signaling due to the elevated levels of VEGFA in their serum (49). However, VEGFA expression in PanNET tissue often inversely correlates with disease severity. High-grade, poorly differentiated, and advanced tumors frequently exhibit decreased VEGFA levels compared with low-grade tumors (50, 51). This “neuroendocrine paradox” may partly explain our finding of lower VEGFA levels in PanNET metastases in the RNA-Seq data set. These insights suggest a potential need for new antiangiogenic treatments that more effectively target advanced PanNETs.

Preclinical studies have shown that tumor vascular modulation, driven by the concurrent targeting of VEGFA and ANGPT2, contributes to antitumor immunity through the activation and perivascular accumulation of T cells (19, 52). Our work not only builds upon and validates immunostimulation by antiangiogenic therapy but also offers potentially unique and significant insights into the vascular regulation of T cell infiltration into the immunosuppressive microenvironment of liver metastases. While earlier tumor xenograft studies demonstrated the antitumor efficacy of ANGPT2 inhibition in mice (53, 54), we found that the antimetastatic effects of targeting ANGPT2 are abolished under immunodeficient conditions. Our observations indicate that the immune system, specifically CD8^+^ T cells, facilitated the antimetastatic responses to anti-ANGPT2 observed in the liver. These responses may involve conventional cytotoxicity against tumor cells and T cell interactions with endothelial cells regulating endothelial functions (55). The increased expression of CXCL11, a key player in T cell recruitment, following ANGPT2 inhibition, might be a contributing factor to the increased T cell infiltration observed in liver metastases. Notably, our data indicate that ANGPT2 inhibition preferentially promoted the infiltration of T cells, rather than other immune cells, like myeloid cells, into liver metastases and also improved T cell function within tumors. Additionally, our results showed a partial suppression of liver metastatic burden after combined treatment with anti-ANGPT2 and anti-CD4 antibodies. In contrast, CD8^+^ T cell depletion abolished the antimetastatic effect of anti-ANGPT2, underscoring a partial and complementary role of CD4^+^ T cells in the CD8^+^ T cell–mediated antitumor immune response. Furthermore, our results indicate that ANGPT2 blockade may not only make tumor blood vessels more permissive to T cell infiltration, but also enhance T cell activation. The increased proportion of activated (CD69^+^ or granzyme B^+^) CD8^+^ T cells after ANGPT2 blockade suggests the possible effect of ANGPT2 inhibition on the activation and maturation of antigen-presenting cells, which could result in enhanced T cell activation. To gain a comprehensive understanding of the T cell–specific regulation exerted by ANGPT2 in advanced PanNETs, further investigations are warranted.

From a clinical perspective, the prognosis for patients diagnosed with PanNETs can be extremely variable due to heterogeneous inter- and intratumoral biology as well as the often-indolent growth kinetics of the malignant cells. While many patients with localized disease can achieve either complete or long-term remission following surgical resection (56), those who relapse have a dismal prognosis. Among the most robust criteria for prognostic stratification of PanNETs are histological differentiation (well-differentiated vs. poorly differentiated) and pathological grade (based on the quantification of the percentage of cancer cells expressing Ki-67) (57, 58). Although a high-grade (G3; Ki-67 >20%) is associated with a high-risk of relapse, it has limited sensitivity in identifying PanNETs at high risk of metastatic progression (59). Our data demonstrate a strong association among elevated ANGPT2 levels, PanNET metastatic progression, and poor prognosis. This implies that elevated levels of ANGPT2 or other TIE signaling molecules could be leveraged to identify patients with PanNETs with tumors characterized by aggressive clinicopathological features and at high risk of metastasis. While our study focused on nonfunctional PanNETs due to their higher clinical prevalence, future comparative studies are needed to investigate differences in the expression of ANGPT2 and other relevant changes between functional and nonfunctional PanNETs.

Several ANGPT2-specific antibodies have been developed and are currently being tested in combination with other targeted therapies in clinical trials for patients with cancer (60). Our data support the rationale for applying anti-ANGPT2 therapy, especially in advanced disease, which might benefit most from the antimetastatic effects of ANGPT2 inhibition. The insights obtained from this preclinical model of metastatic PanNETs might be applicable to metastatic lesions from a broader spectrum of tumor types, particularly those with poor immunogenicity. Moreover, the profound antimetastatic effect of ANGPT2 suggests the potential effect of targeting ANGPT2 to control resistance or metastatic recurrence following anti-VEGF treatment in metastatic settings. Taken together, our work provides insight into how targeting ANGPT2 can promote immunostimulatory reprogramming, enhancing clinical management of metastatic PanNETs and thus controlling disease progression in the liver.

## Methods

Further information can be found in Supplemental Methods.

### Animal models of PanNETs.

The transgenic mice, RT2;B6 (61), were provided by D. McDonald (Helen Diller Family Comprehensive Cancer Center, University of California, San Francisco, San Francisco, California, USA) and were maintained as a colony at Columbia University Irving Medical Center. RT2;AB6F1 mice were generated by mating female A/J mice with male RIP1-Tag2;C57Bl6 mice (37, 38). F_1_ pups were exclusively used for experimentation. A/J mice were purchased from The Jackson Laboratories. Using RT2;AB6F1 mice, tumor progression and metastatic spread in the liver were studied from 12 to 20 weeks of age. Survival studies began at 15 weeks of age and were monitored until 23 weeks of age in RT2;AB6F1 mice. Early-stage metastasis experiments began at 15 weeks of age, when SV40^+^ colonies resemble micrometastases, and continued until 18 weeks of age. Late-stage metastasis experiments began at 18 weeks of age, when metastatic colonies are well established, and continued to 20 weeks of age, when mortality becomes pronounced. To prevent mortality caused by hypoglycemia from the insulinoma, sugar pellets or 5% (w/v) sucrose water were supplemented in addition to the regular diet beginning at 12 weeks of age in RT2;AB6F1 and RT2;B6 mice.

In experimental metastasis models, which allowed us to study the metastatic process without the confounding influence from primary tumors, the PanNET cell line AJ-5257-1 was injected through the tail vein to model late-stage metastasis in the liver, where PanNET cells preferentially metastasize. AJ-5257-1 cells (1 × 10^6^ cells/mouse) in 100 μL sterile PBS were injected into 8-week-old A/J male mice. Following the cell inoculation, A/J mice were monitored until the experimental endpoint, 7 weeks later, at which point liver tissues were collected after cardiac perfusion. SCID (C.B-*Igh-1^b^*/IcrTac-*Prkdc^scid^*) mice, purchased from Taconic Biosciences, were injected with either 99-3o cells (1 × 10^6^ cells/mouse) or AJ-5257-1 cells (5 × 10^5^ cells/mouse) in 100 μL sterile PBS when mice at 9 weeks of age. After the cell inoculation, these mice were monitored until the experimental endpoint, 6 weeks later, when metastatic liver tissues were collected after perfusion. Mice were housed in a pathogen-free barrier facility on a 12-hour light/dark cycle with unrestricted access to food and water.

### Cell culture.

The PanNET cell lines, 99-3o (derived from RT2;B6 mice) and AJ-5257-1 (derived from RT2;AB6F1 mice), were provided by D. Hanahan (Swiss Institute for Experimental Cancer Research, Lausanne, Switzerland) (38). The 99-3o cell line was cultured in DMEM media supplemented with 10% (v/v) fetal bovine serum while the AJ-5257-1 cells were cultured in DMEM F-12 media containing 10% (v/v) fetal bovine serum, 1% (v/v) Insulin/Transferrin/Selenium (Gibco, 41400-045), 4 μg/mL hydrocortisone, and 5 ng/mL mouse EGF. All cell lines were maintained in a 5% CO_2_ incubator at 37°C and tested negative for mycoplasma.

### Treatments.

Spontaneous tumor-bearing RT2;AB6F1 mice were treated either with an anti-ANGPT2 selective antibody (Regeneron Pharmaceuticals Inc., REGN910, 12.5 mg/kg, i.p.) or with IgG (Regeneron Pharmaceuticals Inc., REGN1945, 12.5 mg/kg, i.p.) twice per week. Treatment duration for early-stage experiments extended for 3 weeks, while late-stage experiments were limited to 2 weeks due to increased mouse mortality in the RT2;AB6F1 model. In the experimental metastasis model using AJ-5257-1 cells, treatment with REGN910 or IgG began 3 weeks after the cell injection and continued for 4 weeks. In the CD8^+^ or CD4^+^ T cell depletion studies, mice were placed in the following treatment groups: IgG (vehicle), anti-ANGPT2 (monotherapy), anti-ANGPT2 plus anti-CD8, and anti-ANGPT2 plus anti-CD4. Anti-CD8 (Bio X Cell, clone 2.43, BE0061, 100 μg/mouse, i.p.) or anti-CD4 (Bio X Cell, clone CK1.5, BE0003-1, 100 μg/mouse, i.p.) antibodies were administered on days –2 and –1 prior to the start of REGN910 treatment (day 0) and subsequently given 3 times per week until the end of the experiment. For the PD-1 inhibition studies, mice were divided into 4 groups: IgG (vehicle), anti-ANGPT2, anti–PD-1, and a combination of anti–PD-1 and anti-ANGPT2. The anti–PD-1 (Bio X Cell, RMP1-14, BP0146, 100 μg/mouse, i.p.) antibody was administered 3 times per week, whereas both IgG and anti-ANGPT2 were administered twice a week. All treatments were continued for a period of 2 weeks.

### Genetic deletion of ANGPT2.

*Angpt2^fl/fl^* (39, 40) mice were provided by G.Y. Koh (Korea Advanced Institute of Science and Technology, Daejeon, South Korea), and VE-cadherin-CreER^T2^ mice were provided by R. Adams (Max Planck Institute, Munich, Germany) (41). *Angpt2^iΔEC^* mice were created by mating *Angpt2^fl/fl^* mice with VE-cadherin-CreER^T2^ mice to produce an endothelial cell–specific CreER^T2^ mouse model, enabling conditional deletion of *Angpt2* (*Angpt2^iΔEC^*) upon tamoxifen treatment. In *Angpt2^iΔEC^* mice, *Angpt2* was deleted using the inducible Cre-*loxP* system. Tamoxifen (MilliporeSigma, T5648, 20 mg/mL in corn oil, i.p.) was administered daily for 5 days, beginning at 8 weeks of age in *Angpt2^fl/fl^* mice or control mice lacking VE-cadherin-CreER^T2^ among the littermates. Knockout was confirmed using immunofluorescence analysis of ANGPT2 expression in collected tissues after the conclusion of the experiment and perfusion. AJ-5257-1 cells (1 × 10^6^ cells/mouse) in 100 μL sterile PBS were injected through the tail vein into *Angpt2^iΔEC^* mice at 10 weeks of age.

### Tissue preparation and immunohistochemistry.

At the experimental endpoint, mice were anesthetized with ketamine (90–100 mg/kg) and xylazine (10–17 mg/kg), administered i.p. They were then perfused through the left ventricle with 1% paraformaldehyde (PFA) in 1× PBS for 2 minutes. Following cardiac perfusion, the pancreas and liver were removed, and the left and right median lobes of the liver were isolated and sectioned into 5 pieces (3 pieces from the left lobe and 2 pieces from the right median lobe). Pancreatic and liver tissue were fixed with 1% PFA for 1 hour at 4°C, followed by overnight storage in a 30% w/v sucrose solution in PBS at 4°C. For sectioning with a cryostat, tissues were embedded and frozen in Optimal Cutting Temperature media (Thermo Fisher Scientific, 23-730-571) and sectioned into 50 μm slices (Leica, CM1850). For sectioning with a vibratome, tissues were dehydrated in PBS for 1 hour at 4°C and then sectioned at a 60 μm thickness using a vibrating microtome (Leica, VT1000S). Cryosections were rinsed with PBS containing 0.3% Triton X-100 (PBST) and blocked with 5% normal donkey or goat serum for 1 hour at room temperature. Next, tissue sections were incubated overnight at room temperature with the following primary antibodies: SV40 T antigen (Santa Cruz, SC20800), VEGFR2 (R&D Systems, AF644), CD31 (Thermo Fisher Scientific, MA3105), TIE2 (Regeneron Pharmaceuticals Inc., human monoclonal, REGN1376), ANGPT2 (Regeneron Pharmaceuticals Inc., human monoclonal, REGN910), CD8 (Abcam, ab217344; Invitrogen, MA5-14548), CD4 (Bio-Rad Laboratories, MCA4635), fibrin(ogen) (Dako, A0080), cleaved caspase-3 (Cell Signaling, 9579), claudin-5 (Lifetech, 341600), VE-cadherin (BD Biosciences, 555289; R&D Systems, AF938), and desmin (MilliporeSigma, AB907). After the overnight staining with primary antibodies, sections were rinsed with PBST and incubated with secondary antibodies for 4 hours at room temperature. Nuclei were stained with DAPI (MilliporeSigma, D9542) for 10 minutes at room temperature, and tissues were then mounted using Fluoromount-G media (Invitrogen, 00-4958-02).

### RNA-Seq analysis of human tissues.

We compared 119 healthy livers and 171 healthy pancreas samples from the GTEx database with 30 liver metastases and 83 primary tumors from patients with PanNETs (36). This analysis was conducted using weighted limma-voom (62, 63) in the R/Bioconductor (64, 65) statistical computing environment. Samples were normalized using Smooth Quantile Normalization (66). We focused on genes known to be involved in angiogenic processes.

### Patient samples.

Archived frozen tissue samples were obtained from the tissue bank at the Herbert Irving Comprehensive Cancer Center (HICCC). Histology services, including sectioning, H&E staining, and histopathological interpretation, were performed by the Histology Service at HICCC. Patient plasma samples were also obtained from the tissue bank at the HICCC. These samples were collected in EDTA tubes and centrifuged at 4°C for 20 minutes at 1,700*g* within 2 hours of collection. Subsequently, the samples were stored at –80°C.

### Flow cytometry.

Tumors were minced using razor blades and dissociated into single-cell suspensions by incubating tumors in digestion medium composed of FACS buffer (1× PBS+2% FBS), 0.1% collagenase IV (Worthington, LS004188), and 10U/mL DNase type I (MilliporeSigma, D4527-20KU) for 30 minutes at 37°C with constant shaking. Following digestion, cell suspensions were filtered using a cell strainer (70 μm) to remove cell clusters. Red blood cells (RBCs) were lysed using incubating cell suspensions inside RBC lysis buffer (eBioscience, 00-4300-54). Single-cell suspensions derived from tumors were blocked with rat anti-mouse FcγIII/II receptor (CD16/CD32) blocking antibodies (Fc-Block, BioLegend, 156604) and stained with live/dead cell-exclusion dye (Zombie Yellow dye; BioLegend, 77168). The cells were then incubated for 30 minutes with the following fluorophore-conjugated antibodies from BioLegend: CD45.2 (clone 30-F11, 103107), TCRβ (clone H57-597, 109229), CD206 (clone C068C2, 141731), CD8α (clone 53-6.7, 100741), CD4 (clone GK1.5, 100451), NK1.1 (clone PK136, 108749), CD69 (clone H1.2F3, 104507), CD44 (clone IM7, 103011), CD62L (clone MEL-14, 104417), CD11b (clone M1/70, 101222), F4/80 (clone BM8, 123161), CD11c (clone N418, 117335), MHC II (clone M5/114.15.2, 107621), Granzyme B (clone GB11, 515407), Foxp3 (clone MF-14, 126421), Ly6C (clone HK1.4, 128007), and Ly6G (clone 1A8, 127651). TIE2 (clone TEK4, Invitrogen, 12-5987-82) was also used for the cell staining. Cells were subsequently washed, resuspended in FACS buffer, and analyzed using the Novocyte Quanteon flow cytometry system (Agilent). Flow cytometry analyses of cell populations were performed using FlowJo software version 10.8.1.

### Morphometric measurements.

All images were taken using an Axio Observer 7 microscope with Apotome2 (Zeiss) using either a 10× objective (0.45 NA) or 20× objective (0.8 NA), employing 1-by-1 or 2-by-2-pixel binning, respectively. Image analysis was performed using MATLAB (version 9.7.0.1216025), ZEN 3.0 (version 3.0.79.0000), and ImageJ software (NIH; version 1.53k). The liver metastatic burden was measured using a custom MATLAB script by creating a mask of the whole tissue area via thresholding and then subtracting a mask of the SV40^+^ tumor colonies, allowing us to determine the percentage area of tumor metastasis. The primary pancreatic tumor burden was measured using the Region tool in ZEN 3.0. Vascular density within metastatic colonies was measured in ImageJ as the percentage of the VEGFR2^+^ vessel area relative to the total tumor area. Tumor apoptosis was measured as the percentage of the area exhibiting positive cleaved caspase-3 immunoreactivity relative to the total tumor area. Metrics for ANGPT2, vascular leakage (fibrin), and vascular stability (desmin, VE-cadherin, claudin-5) were measured in ImageJ by dividing the positive vessel by the VEGFR2^+^ or CD31^+^ tumor vessel area, and these results were also presented as percentages. CD8^+^ or CD4^+^ T cell infiltration into liver metastases was measured as the CD8^+^ or CD4^+^ T cell count per mm^2^. For primary tumors, CD8^+^ T cell infiltration was measured as the CD8^+^ T cell count per mm^2^ either in the tumor periphery (<500 μm from tumor boundary) or the tumor core (>500 μm from tumor boundary).

### Monitoring liver metastasis by micro-CT.

Liver metastases were monitored using micro-CT scans of A/J mice injected with AJ-5257-1 cells (1 × 10^6^ cells/mouse). Micro-CT scans were obtained using a Quantum FX micro-CT scanner (Perkin Elmer) and ExiTron nano 6000 CT contrast agent (100 μL/mouse, Miltenyi Biotec, 130-095-146). Mice were anesthetized with isoflurane for the duration of the scan. Baseline CT scans were taken 3 weeks after cell injection and 1 day prior to the start of treatment. Subsequent imaging was performed once a week for 4 weeks, ending 7 weeks after cell inoculation.

### Statistics.

Statistical analyses were performed using GraphPad Prism (version 9.3.1.471). Data are presented as mean ± SEM. Differences between means were compared by a 2-tailed, unpaired parametric *t* test for 2 groups or a 1-way ANOVA followed by Tukey’s multiple comparison test for multiple groups, unless otherwise noted. Survival curves were evaluated using the Kaplan-Meier method, and statistical differences were analyzed using the log-rank (Mantel-Cox) test. Statistical tests are indicated in the figure legends. *P* values indicating statistical significance are presented in figures (*P* ≥ 0.05 not labeled). *P* values of less than 0.05 were considered significant.

### Study approval.

All mouse experiments were conducted in compliance with the Institutional Animal Care and Use Committee guidelines of Columbia University Irving Medical Center and were approved by the Institute of Comparative Medicine at Columbia University Irving Medical Center.

### Data availability.

The data that support the findings of this study are available within the manuscript and its supplemental materials. Values for all data points in graphs are reported in the Supporting Data Values file. All data accessed from external sources and prior publications have been referenced in the manuscript and its supplemental materials. The bulk RNA-Seq data analyzed in this study were obtained from the GTEx database (36). All other raw data are available upon request from the corresponding author.

## Author contributions

EL, SO, and AL designed and performed experiments and analyzed the data. HP performed flow cytometry analyses for immune phenotyping using PanNET mice. JV, SK, AS, and SH performed imaging analysis and conducted additional in vivo experiments. SC performed qPCR and survival experiments in mice. RAF conducted RNA-Seq analysis of human tissues. HR provided human tissues and performed histologic analyses. TF provided intellectual input for study design. HWY provided guidance on tissue imaging and assisted with data analysis. GT provided reagents/materials through Regeneron Pharmaceuticals Inc. EL, SO, AL, and MK wrote and revised the manuscript. MK developed the concepts and directed the research.

## Supplementary Material

Supplemental data

Supporting data values

## Version 1 10/16/2023

Electronic publication
