# Supplementary material for: Angiopoietin-2 blockade suppresses growth of liver metastases from pancreatic neuroendocrine tumors by promoting T cell recruitment
Source: J Clin Invest. 2023 Oct 16;133(20):e167994. doi: 10.1172/JCI167994 (PMC10575726; doi:10.1172/JCI167994)
Supplement: Supplemental data [file jci-133-167994-s079.pdf]

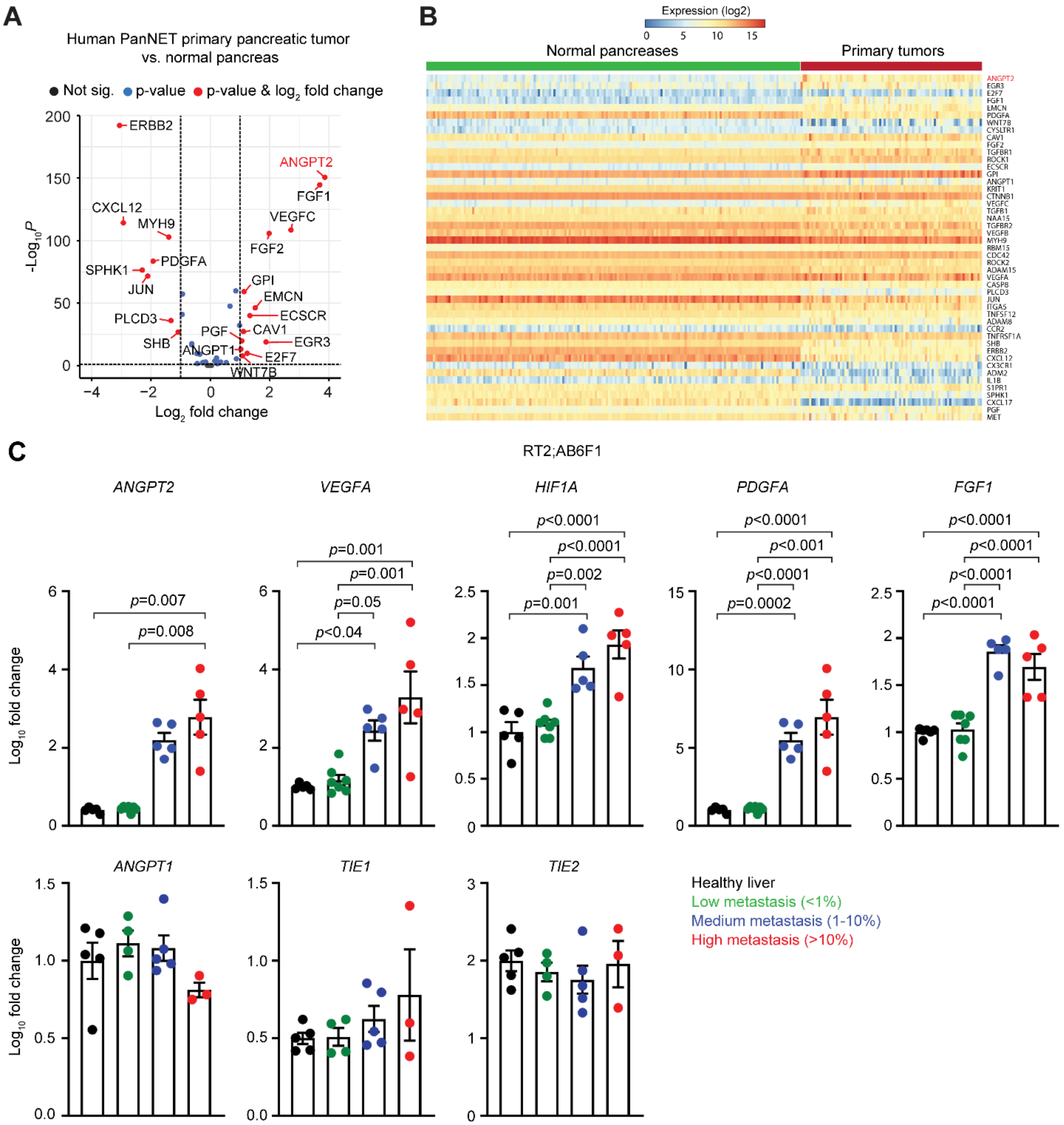

Supplemental Figure 1. Gene expression changes in human PanNET primary tumors and liver metastases from RT2;AB6F1 mice. (**A** and **B**) A volcano plot (A) and heat map (B) from RNA-seq transcriptome analyses of differentially expressed angiogenesis-related genes, including ANGPT2 (red), in primary tumors from PanNET patients (n=83) compared to normal human pancreatic tissues (n=171). The horizontal dotted line in the volcano plot (A) represents a false discovery rate (FDR) of 1%; the vertical lines represent the threshold (±2-fold) of the $\log_2$

fold change. The heat map (B) shows normalized RNA-seq data for angiogenesis-related genes (rows) from 254 samples (columns). (**C**) Quantification of RNA expression levels of selected angiogenesis-related genes, including *ANGPT2, VEGFA, HIF1A, PDGFA, FGF1, ANGPT1, TIE1,* and *TIE2*, in healthy liver tissues (n=5) and liver metastases from RT2;AB6F1 mice, stratified by percent area of metastasis: Low <1% (n=7 or 4), Medium = 1%-10% (n=5), and High >10% (n=5 or 3). For (C), each data point indicates an individual mouse. Statistical analysis was performed using a 1-way ANOVA with Tukey's multiple comparisons test.

A

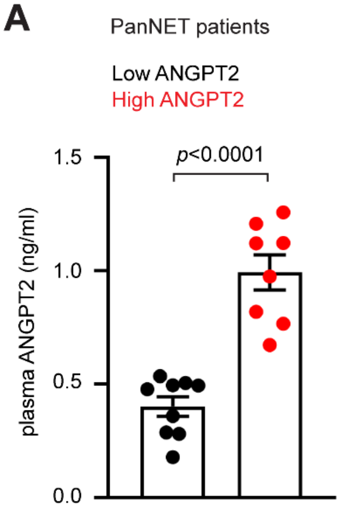

B

		ANGPT2 (ng/ml)			ANGPT2 (ng/ml)
Low ANGPT2 (n=9)	Patient A	0.5352795	High ANGPT2 (n=8)	Patient J	0.673434
	Patient B	0.5060505		Patient K	0.7667685
	Patient C	0.495576		Patient L	0.8198595
	Patient D	0.4940595		Patient M	0.975717
	Patient E	0.4776705		Patient N	1.121913
	Patient F	0.3589935		Patient O	1.1225385
	Patient G	0.287412		Patient P	1.207626
	Patient H	0.2801385		Patient Q	1.2580845
	Patient I	0.177951			

Supplemental Figure 2. Circulating ANGPT2 levels in PanNET patients. (**A** and **B**) Quantification of plasma ANGPT2 levels for each group using an unpaired *t* test: low ANGPT2 (n=9) and high ANGPT2 (n=8) (A), and a table showing the plasma ANGPT2 concentrations for each patient (B).

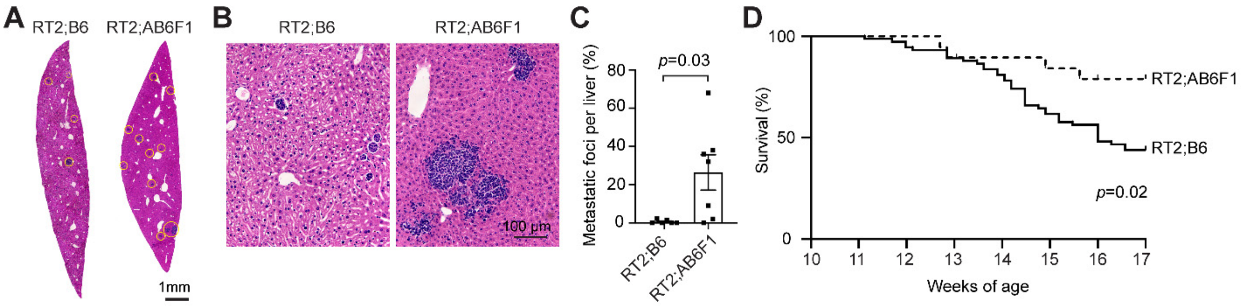

Supplemental Figure 3. Comparison of liver metastasis and survival in RT2;B6 and RT2;AB6F1 mice. (**A-C**) H&E images of whole liver lobe cross-sections, showing metastatic burden highlighted in yellow circles, in RT2;B6 (left) and RT2;AB6F1 (right) mice at 17 weeks of age (A). Magnified images of tissue cross-sections (B) and quantification of the metastatic foci area in the total liver lobe cross-sectional area using an unpaired *t* test (C). (**D**) Kaplan-Meier survival curves representing mortality in RT2;B6 (n=75) and RT2;AB6F1 (n=36) mice up to 17 weeks of age. For C, each data point represents an individual mouse.

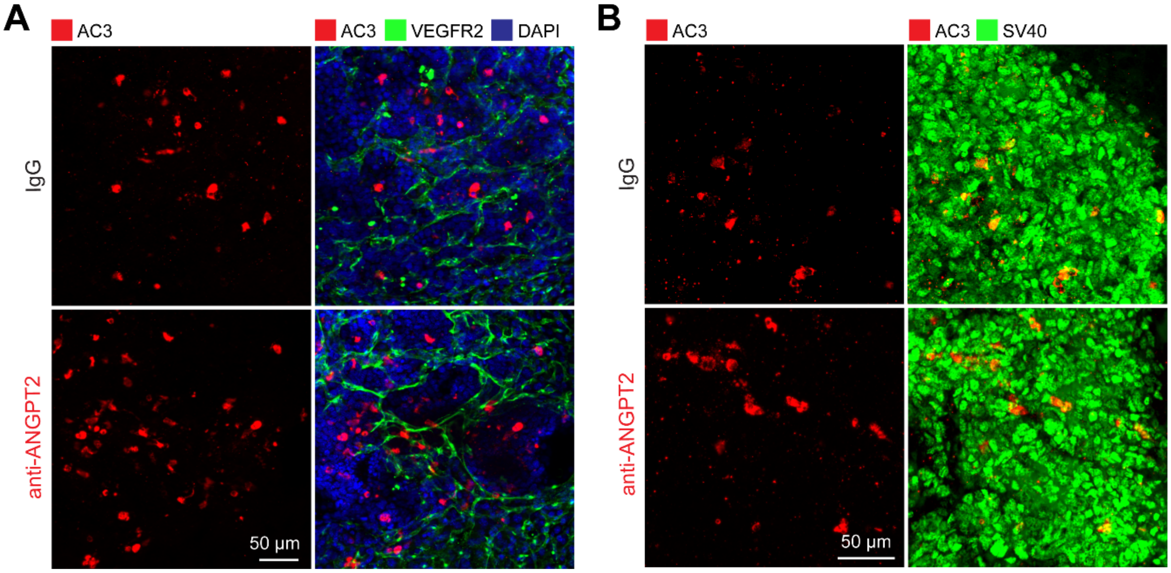

Supplemental Figure 4. Identification of apoptotic tumor cells in liver metastasis from RT2;AB6F1 mice. (**A** and **B)** Representative images of co-staining for activated caspase-3, VEGFR2, and DAPI (A), as well as activated caspase-3 and SV40 T-antigen (B) in metastatic liver tissues from RT2;AB6F1 mice treated with either IgG or anti-ANGPT2.

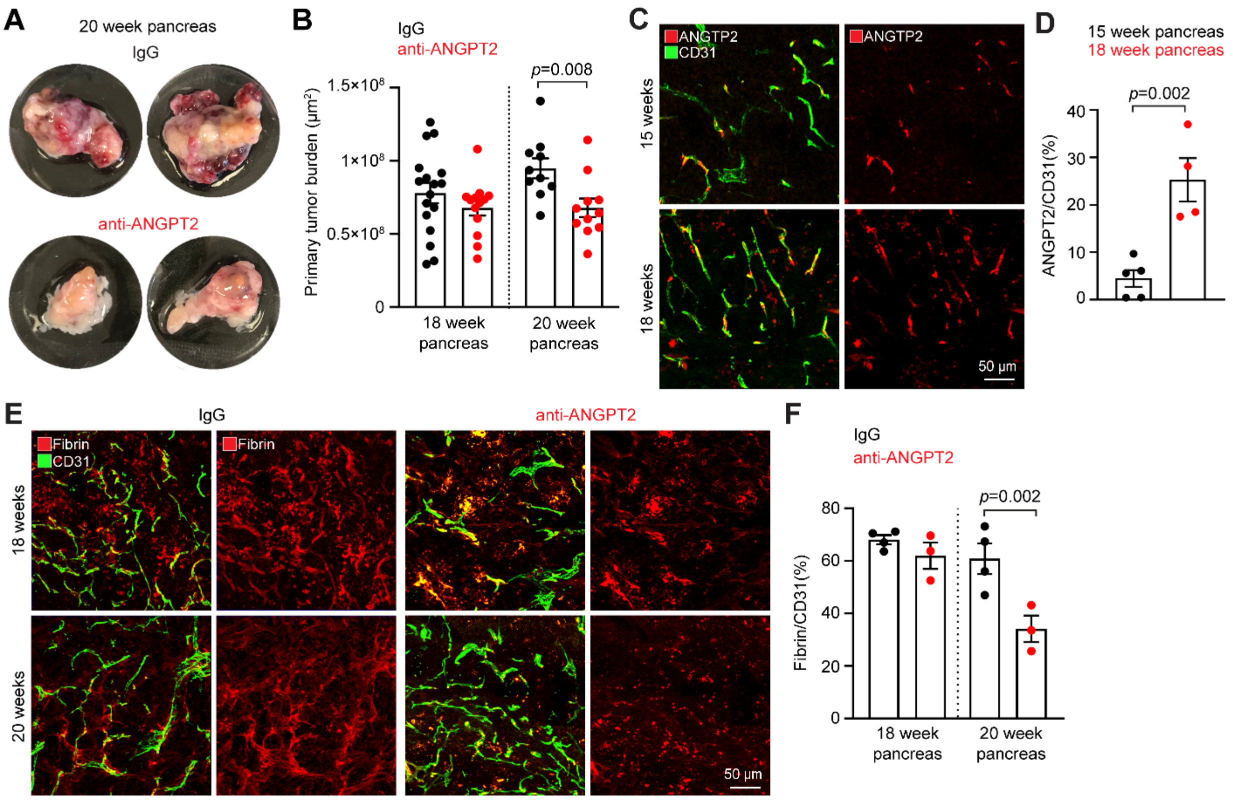

Supplemental Figure 5. Comparison of primary tumor burden from RT2;AB6F1 mice treated with IgG or anti-ANGPT2. (**A**) Macroscopic images of primary pancreatic tumors from RT2;AB6F1 mice treated with either IgG or anti-ANGPT2 at 20 weeks of age (late stage metastasis) after cardiac perfusion. (**B**) Corresponding quantification of primary tumor burden (SV40 T-antigen$^+$) using an unpaired *t* test during early stage metastasis (left) or late stage metastasis (right) experiments, at 18 weeks and 20 weeks of age, respectively. (**C** and **D**) Representative images comparing ANGPT2 expression in blood vessels of primary tumors in RT2;AB6F1 at 15 and 18 weeks of age, respectively (C), and the corresponding quantification of ANGPT2$^+$ vessels using an unpaired *t* test (ANGPT2/CD31, %) (D). (**E** and **F**) Representative images showing vascular leakage, as marked by extravasated fibrin in vessels, in primary tumors in RT2;AB6F1 mice treated with IgG or anti-ANGPT2 at 18 and 20 weeks of age, respectively (E), with the corresponding quantification using an unpaired *t* test (F). For B, D, and F, each data point indicates an individual mouse.

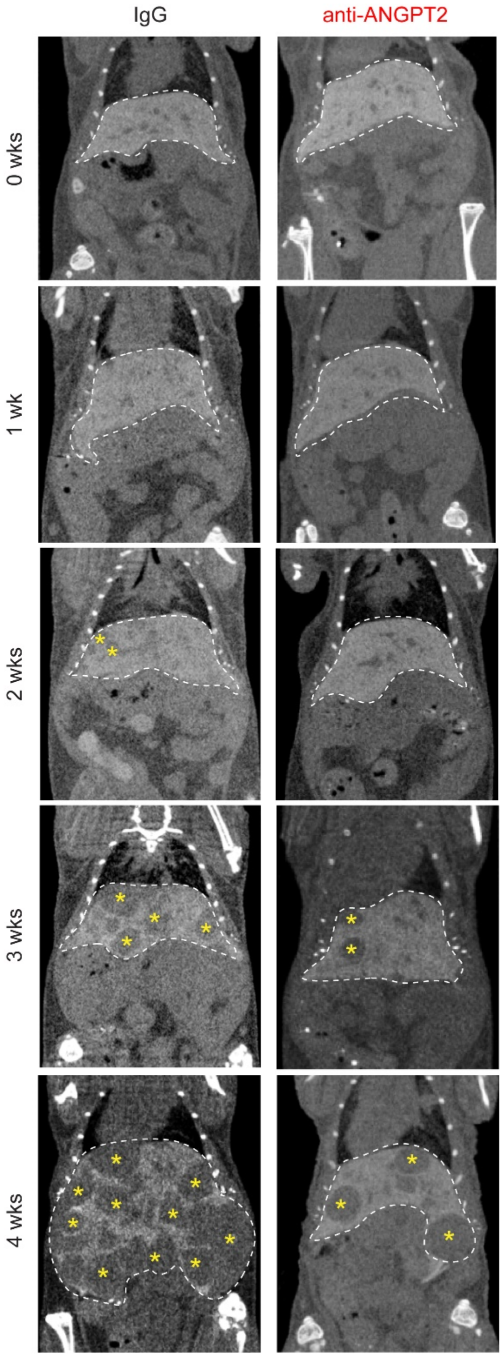

Supplemental Figure 6. Decreased liver metastatic progression by pharmacological ANGPT2 inhibition in AJ-5257-1 mice. Representative micro-CT images of liver metastatic progression (yellow asterisk) in mice injected with AJ-5257-1 cells and subsequently treated with either IgG or anti-ANGPT2 for 4 weeks. White dotted lines indicate the total liver mass in each field of view.

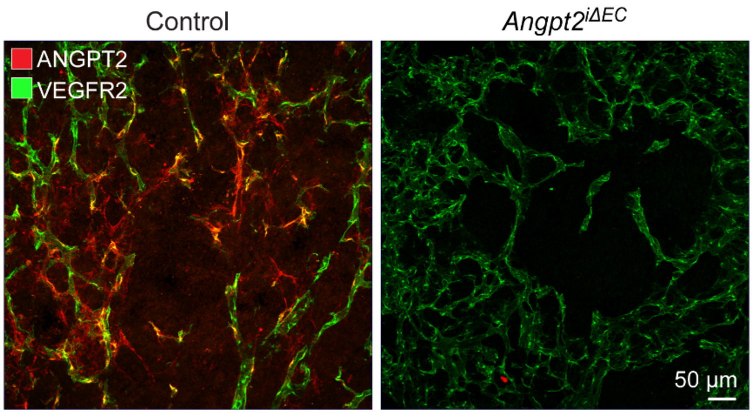

Supplemental Figure 7. Genetically ablated ANGPT2 in liver metastases of *Angpt2*$^{i\Delta EC}$ mice injected with AJ-5257-1 cells. Representative images showing ANGPT2 immunoreactivity (red) in the liver metastasis of control mice (left) but absent ANGPT2 staining in the liver metastasis of *Angpt2*$^{i\Delta EC}$ mice injected with AJ-5257-1 cells.

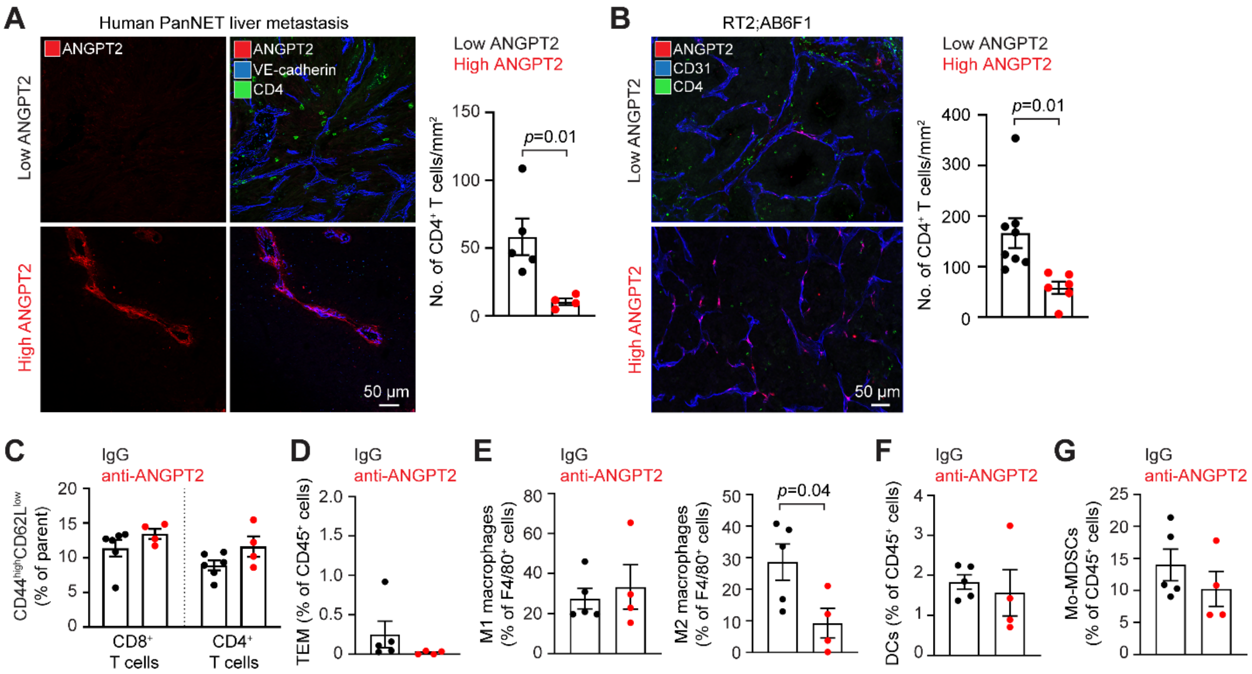

Supplemental Figure 8. Immune regulation of ANGPT2 in PanNET liver metastases. (**A**) Analysis of CD4^{+} T-cell infiltration into the low ANGPT2-expressing (top) or high ANGPT2-expressing (bottom) metastatic livers of PanNET patients and corresponding quantification. (**B**) Analysis of CD4^{+} T-cell infiltration into the liver metastases with low ANGPT2 (top) or high ANGPT2 (bottom) expressions from 18- to 20-week-old RT2;AB6F1 mice and corresponding quantification. (**C**) Flow cytometry analysis showing frequencies of the effector memory (CD44^{high} CD62L^{low}) CD8^{+} and CD4^{+} T cells in the liver metastases of mice inoculated with AJ-5257-1 cells followed by 4 weeks of treatment with IgG or anti-ANGPT2. (**D**-**G**) Flow cytometry analysis showing frequencies of the TIE2-expressing monocytes (TEM, CD11b^{+}TIE2^{+}) (D), M1 (left, CD206^{low}CD11c^{high}) and M2 (right, CD206^{high}CD11c^{low}) macrophages (E), dendritic cells (DCs, MHC II^{+}CD11c^{+}) (F), and Mo-MDSCs (Ly6C^{+}Ly6G^{-}) (G) in the liver metastases in RT2;AB6F1 mice treated with IgG or anti-ANGPT2 at 20 weeks of age. Each data point represents an individual human (A) or mouse (B-G). Statistical analysis was performed using an unpaired *t* test for A-G.

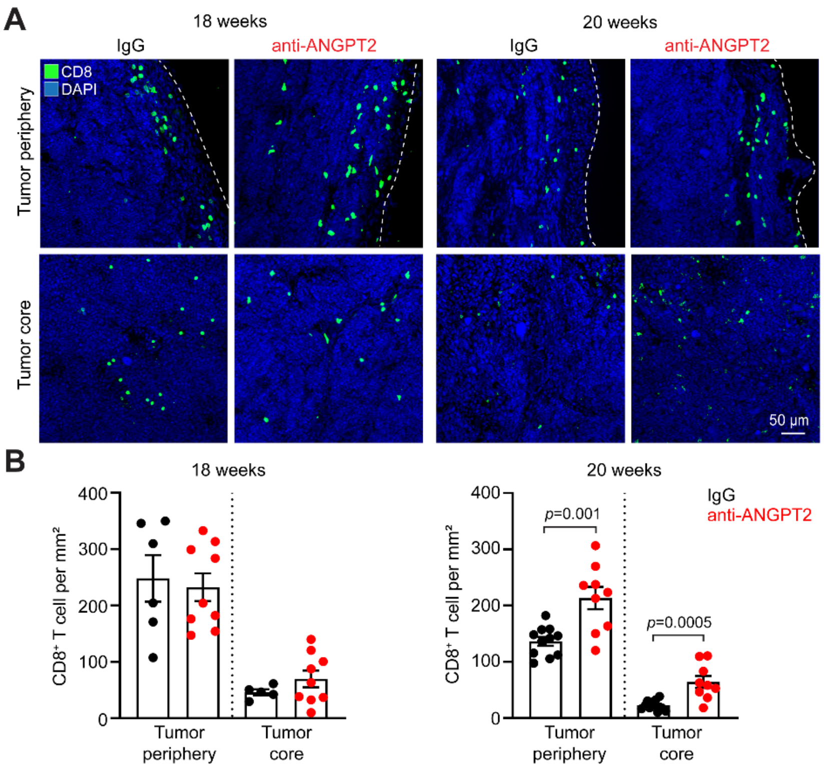

Supplemental Figure 9. CD8$^+$ T-cell infiltration into primary tumors of RT2;AB6F1 mice. (**A** and **B**) Representative images showing CD8$^+$ T cells in both the tumor periphery (< 500 um from tumor boundary) and the tumor core (> 500 um from tumor boundary) of primary tumors from RT2;AB6F1 mice treated with IgG or anti-ANGPT2 until 18 weeks and 20 weeks of age, respectively, and the corresponding quantification of CD8$^+$ T cells with an unpaired *t* test (B). In B, each data point indicates an individual area either at the tumor periphery or the tumor core of each sample. All graphs contain datapoints from at least four individual mice.

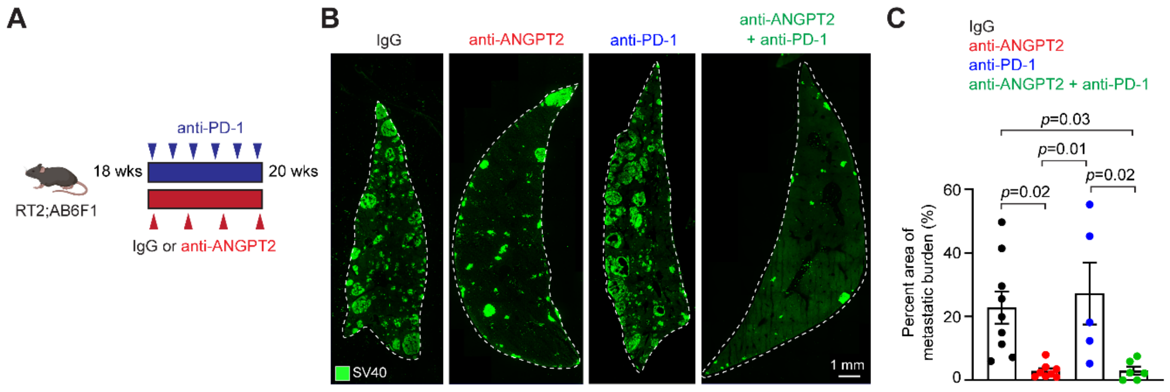

Supplemental Figure 10. A combination therapy of anti-ANGPT2 and anti-PD-1 in liver metastases of RT2;AB6F1 mice. (**A**) Experimental timeline for testing the efficacy of combination therapy of anti-ANGPT2 and anti-PD-1 in RT2;AB6F1 mice. Mice were treated for two weeks with IgG, anti-ANGPT2, anti-PD-1, or anti-ANGPT2 plus anti-PD-1 beginning at 18 weeks of age. (**B** and **C**) Cross-sections of whole liver lobes showing metastatic burden in RT2;AB6F1 mice treated with IgG, anti-ANGPT2, anti-PD-1, or a combination of anti-ANGPT2 and anti-PD-1 (B) and the quantification of SV40 T-antigen^{+} metastatic burden using a 1-way ANOVA with Tukey's multiple comparisons test (C). For C, each data point represents an individual mouse.

Supplemental Methods

Genotyping. AccuStart II Mouse Genotyping Kit (QuantaBio) was used for DNA extraction from RIP1-Tag2 mouse tail snips. We used primers (see Supplemental Resource Tables) and DreamTaq Green PCR Master Mix (Thermo Fisher Scientific, K1081) for detection of the SV40 large T-antigen to identify pups positive for the oncogene. DNA amplification conditions are as follows: 1) 95°C for 2 minutes, 2) 35 cycles of 95°C for 30 seconds, 56°C for 30 seconds, and 72°C for 1 minute, 3) 72°C for 5 minutes. For *Angpt2*$^{i\Delta EC}$ mice, tail snips were digested, and DNA was extracted with DirectPCR Lysis reagent (Viagen, 102-T) and Proteinase K solution (Viagen, 501-PK). We used primers (see Supplemental Resource Tables) along with DreamTaq Green PCR Master Mix for identifying VE-cadherin-CreER^{T2} positive mice. DNA amplification conditions are as follows: 1) 95°C for 5 minutes, 2) 39 cycles of 95°C for 30 seconds, 60°C for 30 seconds, and 72°C for 40 seconds, 3) 72°C for 2 minutes. We used the ProFlex 3x32-Well PCR System (Thermo Fisher Scientific, 4484073) for all thermal cycling.

Secondary antibodies used for immunohistochemistry. Alexa Fluor 488/594/647 Donkey Anti-Goat IgG (705-545-147, 705-585-147, 705-605-147), Alexa Fluor 488/594 Donkey Anti-Rabbit IgG (711-585-152, 711-605-152), Alexa Fluor 594 Donkey Anti-Rat IgG (712-585-150), Alexa Fluor 594 Donkey Anti-Human IgG (709-585-149), and Alexa Fluor 594/647 Goat Anti-Hamster IgG (127-585-160, 127-605-160) from Jackson ImmunoResearch were used.

RNA extraction and qPCR analysis. Approximately 30 mg of liver tissue with a representative metastatic tumor burden was taken from the left median lobe. The selected tissue was isolated, snap-frozen in liquid nitrogen, and stored at –80°C until further use. Total RNA was extracted

using TRIzol (Invitrogen, 15596026) or a RNeasy plus mini kit (Qiagen, 74134), then quantified using NanoDrop One (Thermo Fisher Scientific), and reverse transcription was performed with 1 μg RNA in a 20 μL volume using qScript cDNA Synthesis Kit (QuantaBio, 95048–100). qPCR analyses were performed using PerfeCTa SYBR Green FastMix (QuantaBio, 95074–012) and a QuantStudio 5 Real-Time PCR System (Applied Biosystems). Relative gene expression levels were calculated using the threshold cycle ($2^{-\Delta\Delta CT}$) method and normalized to *Gapdh*. The qPCR primer sequences used for SYBR Green–based qPCR assays are included in the Supplemental Resource Tables.

Supplemental Resource Tables

IHC Antibodies

Target Antigen	Vendor or Source	Catalog Number	Lot Number
Hamster CD31	Thermo Fisher Scientific	MA3105	RF236743
Human ANGPT2	Regeneron Pharmaceuticals Inc.	REGN910	L93
Goat VEGFR2	R&D Systems	AF644	
Human TIE2	Regeneron Pharmaceuticals Inc.	REGN1376	
Rabbit CD8α	Abcam	Ab217344	GR3366917-6
Rat CD4	Bio-Rad Laboratories	MCA4635	1609
Rabbit Cleaved caspase-3	Cell Signaling	9579S	1
Rabbit Claudin-5	Lifetech	34-1600	
Rat VE-cadherin	BD Biosciences	555289	9148638
Rabbit Desmin	Millipore Sigma	AB907	3288723
Rabbit Fibrin(ogen)	Dako	A0080	20061286
Rabbit SV40 T Ag	Santa Cruz Biotechnology	SC20800	G0113
Alexa Fluor 488 SV40 T Ag	Santa Cruz Biotechnology	sc-147 AF488	L1316

Primers for Genotyping

Target	Sequence
SV40 large T-Antigen Forward	5’ - ACTTTGGAGGCTTCTCGGGAT - 3’
SV40 large T-Antigen Reverse	5’ - GGTGTAAATAGCAAAGCAAGCA - 3’

Angpt2 floxed Forward	5’ - AAGCTTGCCATGTCCAAGCTC - 3’
Angpt2 floxed Reverse	5’ - TGATCTTACAGTGCCCACGCAG - 3’
VEcad-creER^{T2} Forward	5’ - AAGTTCATCTGCACCACCG - 3’
VEcad-creER^{T2} Reverse	5’ - TCCTTGAAGAAGATGGTGCG - 3’

Primers for qPCR

**Target**	**Sequence**
Angpt1 Forward	5’ - ACAACACCAACGCTCTGCAA - 3’
Angpt1 Reverse	5’ - TGGCCGTGTGGTTTTGAAC - 3’
Angpt2 Forward	5’ - AAACCACCT TCAGAGACTGTGC - 3’
Angpt2 Reverse	5’ - CATGTCACAGTAGGCCTTGATCT - 3’
Vegfa Forward	5’ - TTACTGCTGTACCTCCACC - 3’
Vegfa Reverse	5’ - ACAGGACGGCTTGAAGATG - 3’
Pdgfa Forward	5’ - CTGGCTCGAAGTCAGATCCACA - 3’
Pdgfa Reverse	5’ - GACTTGTCTCCAAGGCATCCTC - 3’
Fgf1 Forward	5’ - CCAAGGAAACGTCCACAGTCAG - 3’
Fgf1 Reverse	5’ - ACGGCTGAAGACATCCTGTCTC - 3’
Hif1a Forward	5’ - CCTGCACTGAATCAAGAGGTTGC - 3’
Hif1a Reverse	5’ - CCATCAGAAGGACTTGCTGGCT - 3’
Gapdh Forward	5’ - CCTCGTCCCGTAGACAAAATG - 3’
Gapdh Reverse	5’ - TCTCCACTTTGCCACTGCAA - 3’
CXCL9 Forward	5’ - CCTAGTGATAAGGAATGCACGATG - 3’
CXCL9 Reverse	5’ - CTAGGCAGGTTTGATCTCCGTTC - 3’
CXCL10 Forward	5’ - ATCATCCCTGCGAGCCTATCCT - 3’
CXCL10 Reverse	5’ - GACCTTTTTTGGCTAAACGCTTTC - 3’
CXCL11 Forward	5’ - CCGAGTAACGGCTGCGACAAAG - 3’
CXCL11 Reverse	5’ - CCTGCATTATGAGGCGAGCTTG - 3’
ICAM-1 Forward	5’ - AAACCAGACCCTGGAACTGCAC - 3’
ICAM-1 Reverse	5’ - GCCTGGCATTTCAGAGTCTGCT - 3’
VCAM-1 Forward	5’ - GCTATGAGGATGGAAGACTCTGG - 3’
VCAM-1 Reverse	5’ - ACTTGTGCAGCCACCTGAGATC - 3’

All primers were purchased from Integrated DNA Technologies.
